# Solvent Effects in Biomass-Derived Activated Carbons: New Insights for Their Doping/Functionalization toward Potential Hydrogen Storage Applications

**DOI:** 10.1021/acs.langmuir.5c00711

**Published:** 2025-05-14

**Authors:** Alessia MARINO, Carlo POSELLE BONAVENTURA, Sara SCIARRETTA, Giuseppe CONTE, Chiara PELOSI, Andrea LAZZARINI, Alfredo ALOISE, Celia DUCE, Luca BERNAZZANI, Alfonso POLICICCHIO, Marcello CRUCIANELLI

**Affiliations:** 1 Department of Physical and Chemical Sciences, 201850 University of L’Aquila, Via Vetoio, L’Aquila I-67100, Italy; 2 Consorzio Interuniversitario Nazionale per la Scienza e Tecnologia dei Materiali (INSTM), Via Giuseppe Giusti 9, Firenze I-50121, Italy; 3 Department of Physics, 18950 Università della Calabria, Via Pietro Bucci cubo 31C, Arcavacata di Rende (CS) I-87036, Italy; 4 Department of Chemistry and Industrial Chemistry, 154932 University of Pisa, Via G. Moruzzi 13, Pisa I-56124, Italy; 5 CNISM - Consorzio Nazionale Interuniversitario per le Scienze Fisiche della Materia, Via della Vasca Navale 84, Roma I-00146, Italy; 6 Consiglio Nazionale delle Ricerche, Istituto di Nanotecnologia (Nanotec)-UoS Cosenza, Via Ponte P. Bucci, Cubo 31C, Arcavacata di Rende (CS) I-87036, Italy

**Keywords:** solvent role, textural properties, active carbon, hydrogen uptake, gas storage, microporous structure

## Abstract

Biomass-derived activated carbons play an important role in H_2_ storage applications since their structural and chemical properties can be modulated by adjusting the activating methods and experimental parameters as well as by functionalization with heteroatoms. However, unfavorable reaction conditions are usually required, which may compromise the carbonaceous framework, negatively impacting on the hydrogen storage performance. In this context, this work investigates the potential modification effects of different solvents on activated carbons (ACs) under mild conditions, with a focus on structural and textural rearrangements. ACs were treated, among others, with solvents such as toluene (TOL), tetrahydrofuran (THF), and isopropyl alcohol (IPA) at 353 K for a variable amount of time. Structural and textural analyses revealed that solvents might have a significant impact on the microporosity, chemical functionalization, and specific surface area (*S*_BET_) of ACs, thus potentially affecting their further chemical functionalization. TOL and IPA treatments demonstrated the solvent's role in framework reorganization, enhancing microporosity and storage capacity over reaction time. In contrast, THF exposure led to a decrease in textural properties and thermal stability, attributed to disruptive reaction conditions above the solvent’s boiling point. Furthermore, the presence of atmospheric oxygen was found to induce the formation of oxygenated functional groups in the graphitic carbon structure, which contributed to structural instability even if facilitating the framework reordering during prolonged treatments. Although treated samples exhibited reduced hydrogen uptake compared to the parent AC, selected treatments with toluene and IPA demonstrated promising improvements in adsorption efficiency (i.e., H_2_ uptake/*S*_BET_). This study opens the possibility of an effective biomass-derived AC modification, without the need to employ high-energy-consuming thermal treatments, thus maximizing the potential of greener processes.

## Graphical abstract

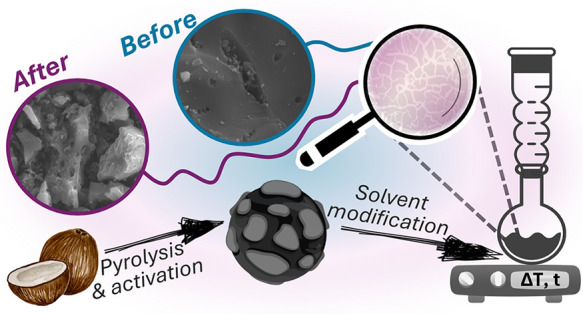

## Introduction

The development of a reliable, efficient, and low-cost storage system is essential for the use of hydrogen (H_2_) as a clean energy carrier since it represents one of the most suitable alternative fuels for the future. Traditional hydrogen storage methods, such as liquefaction and compression, demand substantial energy inputs and present significant safety challenges during transportation.^1^

As a result, research has increasingly focused on alternative storage technologies, particularly on solid-state systems, which can involve chemical or physical matrix-hydrogen sorption–desorption interactions. In the first case, like in the presence of metal hydrides, hydrogen is chemically bound within these systems through ionic, metallic, or covalent bonds. Therefore, if on the one hand this results in high hydrogen uptake capacity, then on the other the actual application is still limited by several challenges, including the non-reversibility or low reversibility of the process, high operating temperatures, and slow H_2_ adsorption/desorption kinetics.^2,3^

To overcome these limitations, physisorption-based systems involving microporous carbonaceous materials have been widely studied, such as carbon nanotubes, nanofibers, and activated carbons, since they allow to operate at lower pressures, offering a complete reversibility and a more rapid kinetics and also leading to relatively high hydrogen uptake.^4^

Among the others, biomass-derived activated carbons (AC) have attracted the attention of the research community due to their sustainability and peculiar features, such as high surface area and chemical stability, which can further be adjusted and customized by selecting the proper matrix,^5–7^ as well as by modifying the activation methods.^8,9^ Moreover, doping AC with heteroatoms, mainly boron (B), nitrogen (N), and sulfur (S), can significantly alter its electronic structure, potentially increasing the interaction between the adsorbed hydrogen and the carbon surface, thus enhancing the storage capacity.^10^ For instance, computational studies have shown that replacing 5–10% of the carbon atoms in a nanoporous structure with B can significantly improve the adsorption energy, with values reaching 10–13.5 kJ/mol, which is attributed to the elongation of the H–H bond and partial charge transfer from the H_2_ σ-bond to the empty localized p_*z*_ orbital of boron.^11–13^

Overall, doped activated carbons are being used for a wide range of applications, such as gas adsorption, water purification, catalysis (both as catalyst or support), and electrochemistry, and therefore, several techniques have been developed for loading various heteroatoms from different sources.

The most used technology for large-scale applications is chemical vapor deposition (CVD),^14,15^ where the heteroatom can be introduced afresh from liquid, solid, or gaseous sources. For instance, Ayala et al. described the use of hot-wall chemical vapor deposition (HW-CVD) to prepare boron-doped ACs by using high vacuum conditions and an ethanol solution of B_2_O_3_,^16^ while Ishii et al. reported methanol and H_3_BO_3_ as precursors, and the hot-filament chemical vapor deposition (HF-CVD) as the selected preparation method.^17^ Shin and Park described an innovative procedure under mild experimental conditions, called “wet procedure”, which involves the functionalization of high surface area graphene oxide by using the BH_3_-THF adduct in THF, at 353 K for 3, 5, or 7 days, without any additional heat treatment.^18^ In a recent study, Asiain-Mira et al. reported an oxidizing treatment of AC in aqueous solution to introduce oxygen as surface functionality, in form of hydroxyl, lactone, and carboxyl groups.^19^ In another case study, ACs were used as support for ionic liquid immobilization, by using methanol as solvent and mild reaction parameters (room temperature for 24 h).^20^ Nitrogen has also been used as a doping agent in electrochemical application, starting from an ethanol solution of melamine or urea and by treating the ACs at 1123 K for 30 min in an inert atmosphere.^21^ Furthermore, sulfur can be covalently bonded in form of S-containing ligands on oxidated ACs by using dimethylformamide as solvent and thionyl chloride as a S source, refluxing the system for 10 h.^22^

What all of these studies have in common is the use of a solvent in the AC doping process. Despite different works having explored the role of the main variables (i.e., temperature, time, doping agents, functional groups, functionalization methods, etc.), to the best of our knowledge, there is still little information about the role and the effects that most of the selected solvent might induce in affecting the surface area on the activated carbon framework. This takes on even greater importance when the gas adsorption is taken into account as an application field, due to the crucial role of the AC surface area in the hydrogen storage capacity.

On these bases, this work aims to investigate how different solvents can affect the activated carbon framework, under mild reaction conditions, with the ultimate goal of providing information about an additional variable to be taken into account during the design of AC chemical functionalization.

Going into detail, the solvent–AC interaction has been studied at three different reaction times (3, 7, and 10 days) by suspending the carbonaceous matrix in the selected solvents, focusing on identifying the most suitable solvent for enhancing the following heteroatom-doping efficiency in ACs, especially for H_2_ storage applications.

## Experimental Section

### Sample Preparation

A defined amount of commercial AC (FILTERCARB PCC, Carbonitalia, 150 mg) was added to 50 mL of the selected solvent in a round-bottom flask without any preliminary treatment. The suspension was sonicated for 3 h at room temperature (RT = 298 K).^18^ Then, the mixture was stirred for designated days (3, 7, or 10) at 353 K. After cooling down, the solid was washed and separated by centrifugation in tetrahydrofuran and, therefore, dried under vacuum overnight for further characterization. The solvent–carbon matrix interaction has been investigated for the following solvents: tetrahydrofuran (THF, Sigma-Aldrich, ≥99.9%), *N*-methyl-2-pyrrolidone (NMP, Sigma-Aldrich, ≥99.0%), 1,2-dimethoxyethane (DME, TCI, >99.0%), toluene (TOL, Sigma-Aldrich, 99,7%), deionized water (W), ethanol (EtOH, Sigma-Aldrich, ≥99.8%), and isopropyl alcohol (IPA, Sigma-Aldrich, ≥99.5%). The obtained samples were named as X_T, in which X represents the acronym of the solvent, and T denotes the treatment time expressed in days (e.g., THF_3).

### Physicochemical Characterization

Textural and porosity features of each sample were investigated by N_2_ adsorption isotherms at 77 K using a Micromeritics ASAP 2460. To eliminate the possible traces of water inside the material, all samples were thermally treated at 373 K for 12 h under vacuum (*P* ≤ 1 × 10^–5^ mbar) before each adsorption test. The surface area (*S*_BET_) was determined using the Brunauer–Emmett–Teller model in the relative pressure range *P*/*P*_0_ from 0.01 to 0.10,^23^ while the micropore area (*S*_micro_) was determined using the t-plot method.^24^ Moreover, the total pore volume (*V*_TOT_) was calculated from the N_2_ uptake at a relative pressure of *P*/*P*_0_ of 0.995. The micropore volume (*V*_micro_) and the pore size distribution (PSD) were calculated on the adsorption branch of the N_2_ isotherm^25^ by using the non-local density functional theory (NLDFT) considering a heterogeneous surface.^26,27^

Thermogravimetry was performed using a Thermobalance (Q5000IR) from TA Instruments equipped with an FT-IR Agilent Technologies spectrophotometer (Cary 640) for evolved gas analysis (EGA). Around 15–20 mg of samples was heated from RT to 1173 K in Pt crucibles, under nitrogen flow (30 mL•min^–1^). The values of mass losses and maximum peak temperature of the derivative curve are reported as the average value of three repeated measures with an error of ±0.2 wt % and ±2 K, respectively. EGA was performed by transferring the gas to the FT-IR spectrometer with a nitrogen flux of 70 mL•min^–1^. FT-IR spectra were acquired every 30 s in the range 600–4000 cm^–1^ with a 4 cm^–1^ width slit. The optical bench was purged with nitrogen, and a background spectrum was recorded just before each analysis. The elemental analyzer CHNS/O PerkinElmer 2400 Series II was used to perform the C, H, N, and S quantification.

Raman spectra of undiluted samples were acquired by means of a Horiba-Jobin Yvon LabRAM spectrometer equipped with an optical microscope (Olympus SLMPLN 20× objective), using an excitation wavelength of 532 nm with a spot size of 1 μm and an irradiation power of 0.1 mW, over 15 mg of sample. Spectra were collected by averaging 20 spectra with a resolution of 4 cm^–1^ in the 4000–400 cm^–1^ range and then normalized with respect to the D peak (∼1350 cm^–1^) and plotted in the 2000–500 cm^–1^ range, where the main features of the samples are present.^28,29^

Infrared spectra were collected using a Bruker VERTEX 70v spectrometer equipped with a DTGS detector. The samples were prepared by thoroughly mixing 250 mg of previously dried KBr powder with 1–2 mg of carbon. The mixture was then pressed into a pellet and measured in transmission mode by averaging 128 spectra with a resolution of 2 cm^–1^ in the 4000–400 cm^–1^ range. Spectra are reported, after scattering background removal, in the 2000–600 cm^–1^ range, where all analyzed samples own the main features.^30^

PHI VersaProbe II (Physical Electronics), equipped with an Al Kα (1486.6 eV) X-ray source, was used for measuring the XPS spectra. The survey spectra were recorded with an analyzer energy path of 117 eV, while the C 1s and O 1s core levels were measured at 23.5 eV passing energy. The X-ray beam size was 100 μm at 25 W. The position of the XPS peaks was referenced to Au metal foil (84.0 eV). XPS peaks were deconvoluted by using MultiPak Data Reduction Software (ULVAC-PHI, Inc.), employing a Shirley background curve. The fitting was applied consistently to each spectrum.

Morphological and structural properties of ACs were observed by using an FEI Quanta 450 ESEM FEG (Bruker) with an accelerating voltage of 15 kV and a working distance of 6.5 mm.

H_2_ adsorption/desorption measurements were performed at RT and 77 K within a pressure range of 0 to 1 bar using the Micromeritics ASAP 2460 porosimeter. Similar to the previously described N_2_ adsorption test, before each measurement, the samples were outgassed overnight at 373 K under vacuum (*P* < 10^–5^ mbar) to remove any weakly bound water inside the samples.

## Results and Discussion

### Structural Analysis

N_2_-isotherm analysis of the treated samples revealed that both solvent type and reaction time affect the textural properties of the AC. As shown in Figure 1 below, all prepared samples exhibit a type I isotherm, typical of microporous materials,^31,32^ but with a quantity of adsorbed gas lower than reference AC and the consequent reduction of *S*_BET_. However, very few samples highlight capillary condensation phenomena (see *P*/*P*_0_ > 0.9), indicative of mesoporosity development.

**Figure 1:**
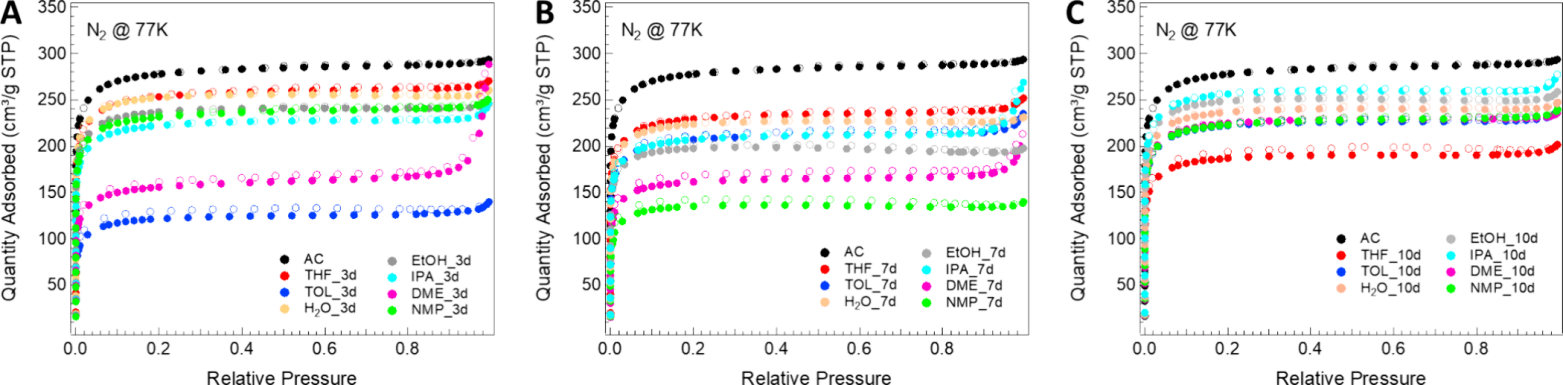
N_2_ adsorption–desorption isotherms of the treated samples at (A) 3 days, (B) 7 days, and (C) 10 days.

In detail, already after 3 days of treatment, a strong drop in the *S*_BET_ of 56 and 44% can be observed when toluene and DME, respectively, were used as solvents, compared to the parent AC. On the contrary, for the same contact time (3 days), only a smaller decrease of *S*_BET_ close to 9–20% was detected using other solvents, indicating a softer interaction with the carbonaceous framework (see Table 1). A similar trend of reduction was recorded by increasing the treatment time up to 7 days, with the only exception of TOL and DME in countertrend with the others, while after 10 days, even if maintaining a *S*_BET_ < 1000 m^2^/g, the differences between the samples are reduced, showing isotherms with similar trends, as well as values of adsorbed N_2_. This suggests that after an initial strong interaction with the solvents, a reorganization of the carbon matrix occurs after a certain contact time. The unique deviation from this general behavior has been represented by samples treated in THF, which showed a gradual decrease in the surface area with increasing reaction time.

**Table 1:** AC Textural Parameters and H_2_ Uptake after Solvent Treatment

**sample**	***S***_**micro**_(m^2^/g)	***S***_**BET**_(m^2^/g)	***V***_**micro**_(cm^3^/g)	***V***_**TOT**_(cm^3^/g)	**H**_**2**_uptake*(mmol/g)	**H**_**2**_uptake* (wt %)
AC	778	1065	0.396	0.405	7.99	1.61
TOL_3d	292	463	0.180	0.195	4.19	0.85
DME_3d	382	593	0.224	0.365	4.42	0.89
NMP_3d	658	891	0.329	0.341	6.54	1.32
THF_3d	699	973	0.380	0.387	7.14	1.44
H_2_O_3d	727	975	0.375	0.375	7.37	1.49
EtOH_3d	675	914	0.353	0.353	6.74	1.36
IPA_3d	603	852	0.332	0.346	6.17	1.24
TOL_7d	560	792	0.303	0.326	5.82	1.17
DME_7d	436	618	0.234	0.282	4.91	0.99
NMP_7d	370	522	0.197	0.197	4.33	0.87
THF_7d	631	878	0.340	0.360	6.44	1.30
H_2_O_7d	638	863	0.331	0.331	6.21	1.25
EtOH_7d	587	768	0.289	0.289	5.63	1.13
IPA_7d	572	796	0.307	0.373	5.92	1.19
TOL_10d	617	855	0.334	0.340	5.86	1.18
DME_10d	631	866	0.337	0.338	6.22	1.25
NMP_10d	625	858	0.336	0.344	6.36	1.28
THF_10d	515	719	0.282	0.286	5.36	1.08
H_2_O_10d	679	908	0.345	0.346	6.29	1.27
EtOH_10d	710	955	0.365	0.365	6.67	1.34
IPA_10d	727	988	0.379	0.389	7.19	1.45

Therefore, although after 7 and 10 days of treatment all N_2_ adsorption isotherms still preserve their type I shape, a considerable change in the surface areas and PSDs can be observed, leading in some cases to a reversal of the trend by increasing the treatment time. As an example, the AC treatments in toluene for 7 and 10 days give rise to higher surface areas, compared to the shorter treatment (TOL_3d); moreover, as shown in Figure 2, the formation of a micropore family with a diameter centered at 0.65 nm occurred at longer contact times, demonstrating the active role of this solvent in rearranging the carbonaceous structure.

**Figure 2:**
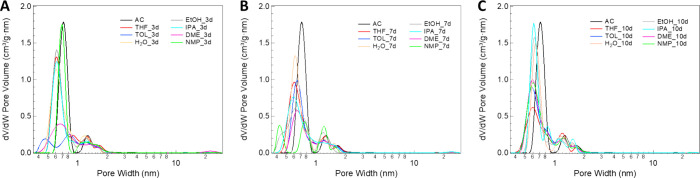
Pore size distribution of the prepared samples at (A) 3 days, (B) 7 days, and (C) 10 days.

A similar trend can be observed in IPA_10d, since a similar drop of *S*_BET_ was detected after 3 and 7 days, compared to the parent AC, while a new increase of both surface area and micropore volume was measured after 10 days of reaction.

Conversely, THF showed the most linear trend by increasing contact time, since at shorter reaction time (3 days), the surface area loss can be considered negligible as well as the total pore volume. Subsequently, the *S*_BET_ value decreases by increasing the reaction time from 7 up to 10 days, at once with the formation of larger micropore families.

Differences among the samples can be easily observed by analyzing the NLDFT PSD reported in Figure 2. In the samples with the lowest *S*_BET_ (TOL_3d and DME_3d), the formation of new families of pores occurred, at the expense of the native micropores with diameter centered at 0.7 nm observed in the AC sample. It is noteworthy that in the presence of ethanol, THF, IPA, and water, the diameter of the main micropore family is shifted to lower values, indicating a new arrangement of the carbonaceous structure.

Overall, although a reduction in *S*_BET_ occurs in the samples, a high degree of microporosity distributed differently between the ultramicropore (<0.7 nm) and supermicropore (0.7–2 nm) regions is maintained, without the development of mesopores (>2 nm). In this regard, some significant differences can be observed among the prepared samples in the presence of toluene, THF, and IPA, as solvents, and 3 and 10 days as treatment times.

Going into details, for the samples treated with toluene, a significant increase in the *S*_BET_ of 84% is observed in TOL_10d, compared to the 3-day sample. At the same time, there is also an improvement in the total pore volume (*V*_TOT_), with a significant increase in the microporous component moving from 0.180 to 0.334 cm^3^/g. The supermicroporous (0.7 nm < *V* < 2 nm) component increases by 26%, while a drastic increase of more than four times is detected in the ultramicroporous (*V* < 0.7 nm) component (see Table 2).

**Table 2:** AC Textural Parameters of Samples Treated with Selected Solvents for 3 and 10 Days

**sample**	***S***_**BET**_ [m^2^/g]	***V***_**<0.7nm**_ [cm^3^/g]	_**0.7nm<**_ ***V***_**<2nm**_ [cm^3^/g]	***V***_**micro**_ [cm^3^/g]	***V***_**meso**_ [cm^3^/g]	***V***_**TOT**_ [cm^3^/g]	***V*** **_micro_/** ***V***_**TOT**_ [%]
AC	1065	0.110	0.286	0.396	0.009	0.405	98
TOL_3d	463	0.037	0.143	0.180	0.015	0.195	92
TOL_10d	854	0.152	0.181	0.334	0.006	0.340	98
IPA_3d	852	0.179	0.153	0.332	0.014	0.346	96
IPA_10d	988	0.210	0.169	0.379	0.010	0.389	97
THF_3d	973	0.190	0.190	0.380	0.007	0.387	98
THF_10d	719	0.114	0.169	0.283	0.004	0.286	99

A similar but less pronounced behavior is observed using the IPA solvent, with an increase in *S*_BET_ of only 15%, correlated with an increase in *V*_TOT_ of about 12%. As the previous analyzed case, in the ultra- and supermicroporosity is observed a growth of 17 and 10%, respectively.

In contrast, a decrease in *S*_BET_ from 973 m^2^/g of the 3-day sample to 719 m^2^/g in the 10-day sample has been measured in the presence of THF as solvent, as well as a decrease of about 27% in *V*_TOT_, while maintaining almost unchanged the micropore ratio *V*_micro_/*V*_TOT_. In the all-analyzed structures, the mesoporous component never exceeds a value of 0.015 cm^3^/g.

On the basis of these peculiar structural data, the abovementioned three solvents (i.e., toluene, tetrahydrofuran, and isopropyl alcohol) at the farthest reaction conditions (i.e., 3 and 10 days) have been selected as the most interesting cases for better understanding how the used solvents may affect the AC rearrangement during the contact time. To this aim, a further characterization has been carried out on the six selected samples and compared to the parent AC.

First, the morphology of the ACs after 3 or 10 days of solvent treatment was investigated through SEM analysis, carried out in various areas of each sample and at different magnifications. Just a slight increase of surface roughness can be observed depending on the carbon–solvent contact time (see Figure 3 for THF-treated samples). However, no significant differences were detected regardless of the solvent type (Figure S1).

**Figure 3:**
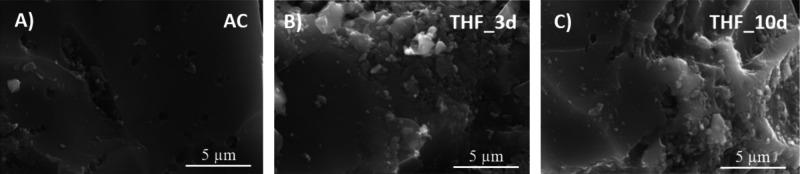
SEM images of (A) parent AC, (B) THF_3d, and (C) THF_10d (magnification: 20,000×).

With a view to assessing possible changes in the carbon and oxygen contents, the elemental composition of the selected ACs was investigated by CHNS/O analysis. However, the results shown in Table S1 (Supporting Information) revealed, also in this case, no significant differences among the samples, regardless of both solvent type and reaction times.

To explore in depth the carbonaceous framework order, the selected ACs were analyzed by Raman spectroscopy, and the obtained spectra are reported in Figure 4. Two bands have been detected in all samples: (i) one around 1330 cm^–1^ identified as D, which is due to A_1g_ vibrational mode, arising when there is a limited lateral dimension of graphene-like platelets, implying a certain short-range order; (ii) a second band, centered at 1590 cm^–1^ (G band), which is related to the E_2g_ mode of sp^2^ aromatic carbon.^33^

**Figure 4:**
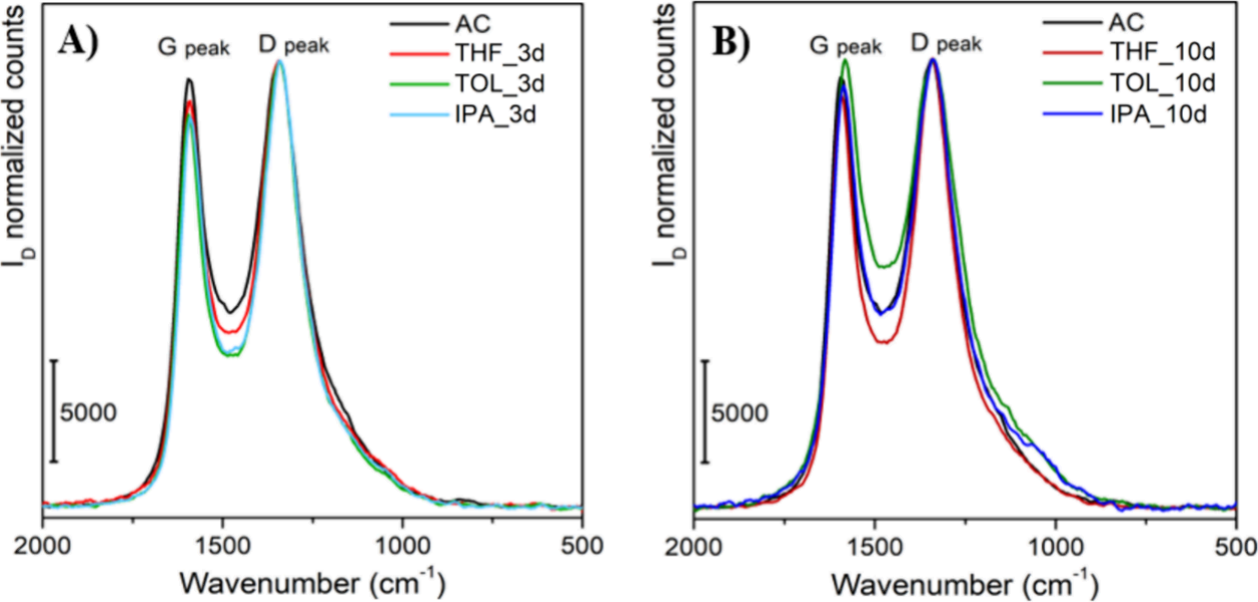
Raman spectra of the treated samples at (A) 3 days and (B) 10 days, compared to the parent material.

The degree of long-range order is thus expressed as the ratio between the intensities of D and G bands (*I*_D_/*I*_G_), and the results concerning the prepared samples are reported in Table 3. For all samples treated either for 3 or 10 days with any solvent, no relevant modifications in the *I*_D_/*I*_G_ ratio can be observed, meaning that there are no big changes in the lateral dimensions of the graphene-like platelets;^34^ however, few small changes that appeared in the *I*_D_/*I*_G_ ratio might give useful insights in understanding the reasons behind the variation in the observed values of surface area and pore volume.

**Table 3:** *I*_D_ /*I*_G_ Ratios from Raman Spectra Acquired on Solvent-Treated Samples

**sample**	***I***_**D**_ **/*I***_**G**_
AC	1.05
THF_3d	1.09
THF_10d	1.10
TOL_3d	1.14
TOL_10d	1.00
IPA_3d	1.14
IPA_10d	1.06

All AC samples treated for 3 days in the different solvents show a slight reduction of the G peak with respect to the parent material (AC), meaning that they have a tendency to introduce structural disorder in the materials when employed in those conditions. Conversely, for samples treated at 10 days, a slight increase of G peak can be observed, with respect to the samples treated at 3 days, indicating a restoration of the structural order in the materials.^35^ A similar trend has been observed in literature for ACs retreated at high temperature, showing a progressive ordering of the structure either with higher temperatures or with longer reaction time.^36^ Another indication of partial ordering of the structure is the red shift of the G band for samples IPA_10d and TOL_10d, shifting to 1585 and 1582 cm^–1^, respectively, moving progressively toward the peak position of crystalline graphite (1580 cm^–1^).^37^ This is associated with a partial tensile stress relief of the aromatic structure, suggesting a slight but progressive ordering of the material.^38–41^ Indeed, ACs are metastable materials; thus, a long exposure to harsh physicochemical environments might tend to push the structure toward its thermodynamic minimum (i.e., graphite-like structure).

The restructuring of the aromatic part of the carbons does not exclude the presence of a minority of other carbonaceous species. In fact, a small band centered at 1470 cm^–1^ (with constant intensity for all the samples) is present, namely, the D3 band. This signal is associated with a statistical distribution of amorphous carbon on the interstitial position of the graphitic islands,^42^ which in our case is not altered by any of the conditions used. Finally (only for IPA_10d and TOL_10d samples), there is the arising of a small band at 1150 cm^–1^, defined as I band, originating from a coexistence of sp^3^- and sp^2^-hybridized phases (the last one in the form of conjugated nonaromatic polyenes).^43^ In particular, this band might originate from the aperture of some aromatic rings at the edges of the graphitic platelets of the carbon materials. This might also have helped the structural stress reduction evidenced by the G peak shift for the same samples.

Aiming to further investigate the interaction between the carbonaceous framework and the used solvents, thermogravimetric analysis coupled with FT-IR was carried out on the selected samples. The TGA/FT-IR profile of parent AC under an inert atmosphere shows a total mass loss of 12.2% when heated up to 1173 K. The loss is mainly due to moisture evaporation that occurs below 373 K, as highlighted by the FT-IR spectrum of the evolved gas, which shows the signals of O–H stretching and H–O–H bending in the ranges 4000–3300 and 2000–1200 cm^–1^, respectively (Figure 5B). The origin of the water released is probably due to the physisorbed atmospheric moisture, while the remaining carbonaceous matrix shows a small, slow, continuous mass loss over the temperature range 420–1173 K (Figure 5A), with no detectable signals in the FT-IR spectra, in agreement with the literature.^44^

**Figure 5:**
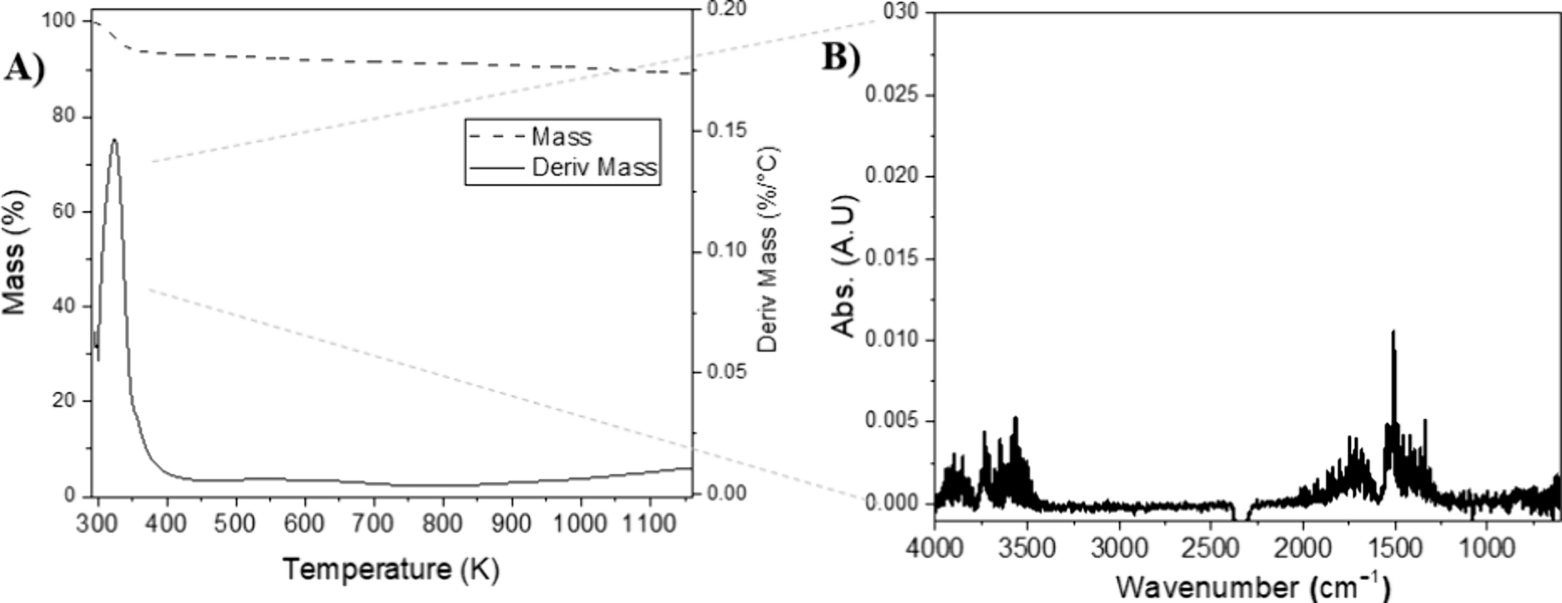
(A) Thermal profile of AC obtained by TGA-FT-IR analysis. (B) FT-IR spectrum of gases evolved during thermal degradation of AC recorded at 320 K.

The analysis of ACs treated for 3 or 10 days in THF, TOL, and IPA provided additional details on the effect of the solvent–carbon matrix interaction. The complete thermograms obtained (Figure S2) show a mass loss of 3–6% below 423 K, which can be attributed to moisture adsorbed during the storage or traces of solvent still present in the samples (FT-IR signals are below the detection limit), despite the drying treatment performed prior to the analysis.

Since the mass loss pattern of all samples below 423 K is similar, even though the entity of the loss is affected by their intrinsic features, the TGA curves were rescaled setting to 100% the dry mass of the sample measured at 423 K. The rescaled thermal profiles are shown in Figure 6, while Table 4 summarizes the normalized mass losses and relative peak temperatures.

**Figure 6:**
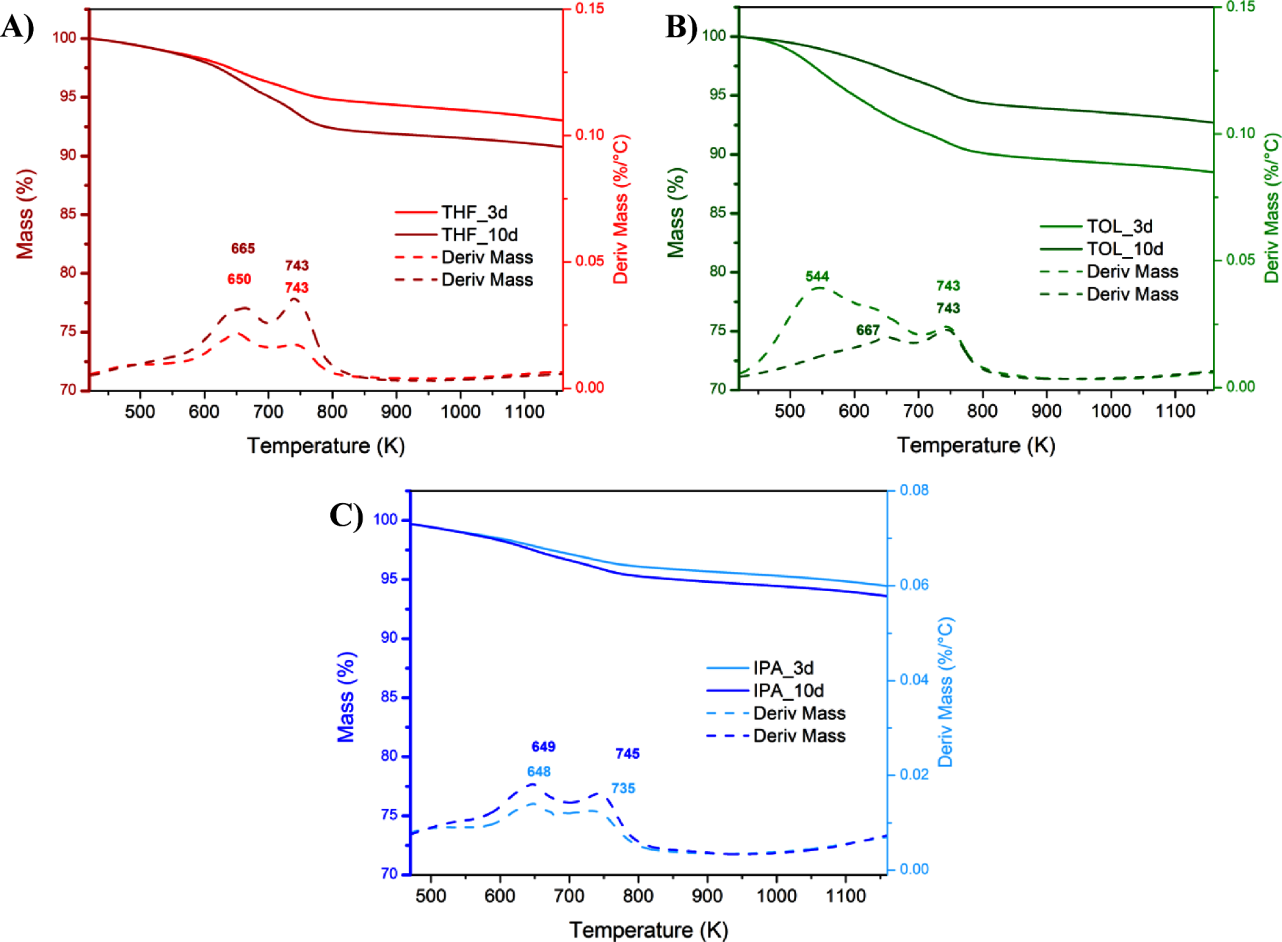
Thermal profiles normalized at 423 K of the samples treated in (A) THF, (B) TOL, and (C) IPA.

**Table 4:** Normalized Mass Loss Obtained by TGA-FT-IR Analyses

**sample**	**mass loss%** (423–573 K)	**mass loss%** (573–873 K)	**mass loss%** (873–1173 K)	**residue** (>1173 K)
AC	1.0	1.4	2.4	95.2
THF_3d	1.5	4.1 (650/743)^a^	1.6	92.8
THF _10d	1.6	6.5 (665/743)^a^	1.3	90.6
TOL_3d	4.0	5.9 (743)^a^	1.4	88.7
TOL_10d	1.4	4.6 (667/743)^a^	1.4	92.6
IPA_3d	1.3	2.9 (648/735)^a^	1.5	94.4
IPA_10d	1.4	3.7 (649/745)^a^	1.5	93.5

a Peak temperatures (K) of the derivative mass loss (DTG).

Samples treated with toluene show the highest improvement in thermal stability with increasing treatment time, as highlighted either by an increase in the final residue at 1173 K (88.7% at 3 days, and 92.6% at 10 days) or by the disappearance of the peak at lower temperatures (544 K) visible in the DTG of the sample TOL_3d. This trend is consistent with the porosity analysis performed by N_2_ physisorption, showing that toluene strongly influences the sample morphology at low treatment time (3 days), while a longer treatment time (10 days) favors the reorganization of the carbonaceous structure, with a consequent increase of the sample thermal stability. The opposite trend is observed in the samples treated with THF: a decrease in the *S*_BET_ with treatment time (from 3 to 10 days) is related to a lower thermal stability, highlighted in the TG analysis by the decrease of the residual mass calculated at 1173 K (92.8% at 3 days, and 90.6% at 10 days). Finally, the samples treated with IPA do not show an evident trend, suggesting that in this case, modifications in *S*_BET_ are too small to induce a clear change in the thermal stability of the samples.

The analysis of the chemical nature of the gases evolved during the thermal degradation by FT-IR spectroscopy made it possible to achieve additional information. The mass loss observed for THF_3d, THF_10d, IPA_3d, and IPA_10d in the range 573–873 K was due to the simultaneous elimination of carbon dioxide (CO_2_) (roto-vibrational stretching mode of the molecule in the gas phase centered at 2350 cm^–1^ and its relative bending mode at 667 cm^–1^) and small aliphatic molecules (C–H stretching in the range 2800–3000 cm^–1^), as shown in Figure 7A and Figure S3. The release of CO_2_ may be an index of the thermal degradation of oxygenated groups formed on the surface of ACs during solvent treatment.^45^ Moreover, a small signal ascribable to the evolution of gaseous CO (roto-vibrational signal at 2153 cm^–1^) appears for temperatures above 1000 K (Figure S3). The sample TOL_3d presents an additional peak on the FT-IR spectra centered at 1739 cm^–1^, due to the carbonyl stretching (see Figure S3). This signal, whose presence is in accordance with the strong change of the AC morphology induced by toluene after 3 days of treatment, disappears when the sample is treated in toluene for 10 days (TOL_10d), and it evolves in a more ordered structure. Unfortunately, such signal is difficult to identify, since a plethora of oxygenated organic species possess an IR feature in such narrow window in the gas phase.^46^

**Figure 7:**
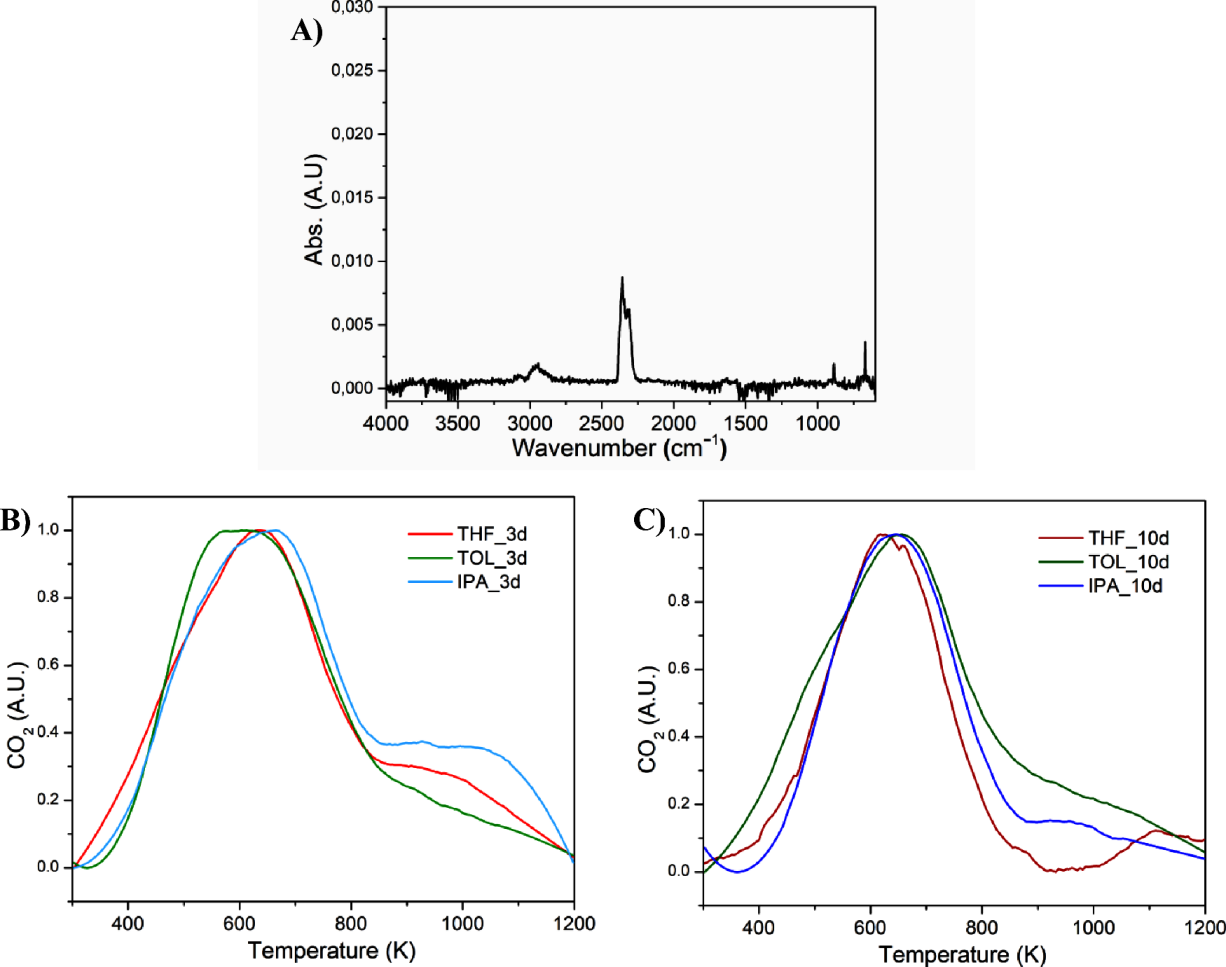
(A) Representative FT-IR spectrum of gas evolved at 640 K for THF_3d; evolution profiles of CO_2_ emission during the thermal scan for the samples treated for (B) 3 days and (C) 10 days (red curve = THF, green curve = TOL, blue curve = IPA).

The identity of the groups present on the AC surfaces after the solvent treatment was studied in more detail by observing the evolution profile of CO_2_ (Figure 7b). In particular, CO_2_ emitted by all ACs treated with THF, TOL, and IPA for 3 days is due mainly to decarboxylation of carboxylic acids present on the surface, which have a degradation temperature in the range 400–800 K. In addition, the presence of anhydrides could also justify the evolution of CO_2_ at temperatures above 800 K, with a relative abundance that decreases going from IPA, to THF, to TOL.^45^ On the other hand, the treatment with the same solvents for 10 days does not alter significantly the profile of CO_2_ evolved up to 800 K, except by small differences in peak temperatures, afterward the trend changes increasing from THF to IPA to TOL, indicating the presence of different functional groups on the AC surface, and therefore confirming a framework rearrangement (see Figure 7C).

Despite the strong absorbing nature of carbon-based materials in the mid-IR spectral region,^47^ FT-IR spectroscopy can be extremely useful to identify the chemical species present in the materials. All the FT-IR analyses performed are reported in Figure 8, and the results are compared with the parent carbon (AC, black curve).

**Figure 8:**
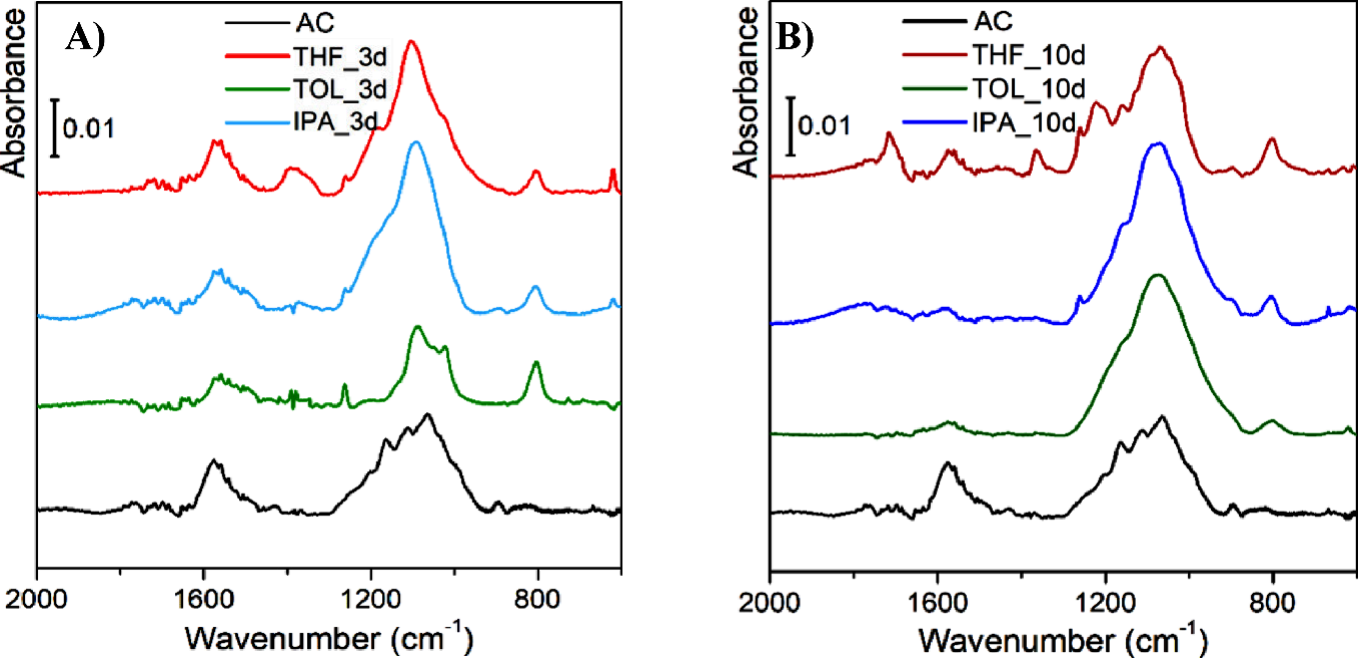
FT-IR spectra of fresh activated carbon (black) and of samples treated in THF (red), TOL (green), or IPA (blue) for (A) 3 days and (B) 10 days.

It is immediately clear that the starting AC sample is a case of a quite well-ordered carbon, testified by the presence of mainly two signals: (i) a band centered at around 1590 cm^–1^, due to the ν­(C=C) vibrational modes of conjugated sp^2^ bonds belonging to graphitic platelets^29^ and (ii) a very broad band between 1300 and 1000 cm^–1^, due to the overlap of several hardly distinguishable absorption signals coming from in-plane C–H bending modes and/or further C–C skeletal vibrations of the graphitic islands.^47^ Furthermore, bands ascribable to the presence of oxygenated groups are almost absent in the AC parent material, as confirmed previously by TGA/FT-IR combined data (Figure 5).

When the samples are exposed to the solvents for 3 days (Figure 8A), the appearance of a new common feature for all the treated samples can be observed, namely, the growth of a signal at 800 cm^–1^ that can be described with the formation of new edges of graphitic platelets, introducing new C–H terminations, of which such vibration represents its out-of-plane bending mode.^48,49^ In the same region, it is also plausible the formation of small aliphatic groups (methyl or ethyl) at the edge of graphitic platelets of the carbon material, contributing in the same spectral region.^46^ This might also explain the observation of small aliphatic groups emerging from TGA. Even if present, the very low intensity of the band demonstrates that such effect is minor, also confirmed by the quite small changes in the Raman spectra (see Figure 4), testifying minimal changes in the structural order.

For samples treated for 3 days, we also observed the rise of two signals at 1370 and 1715 cm^–1^, which can be explained with the introduction of a small amount of oxygenated functionalization, mainly carbonyls from carboxylic groups.^46^ Their introduction may lead to an opening of the terminal rings of the graphitic islands (thus disrupting the sp^2^ structure), with the generation of, respectively, the former signal due to δ_s_(CH_2_) vibration,^50^ and the latter due to ν­(C=O) vibration.^51^

For samples treated for 10 days with the same solvents (Figure 8B), we observed different trends for some of them. Indeed, for ACs modified in the presence of TOL and IPA, the signals ascribed previously to oxygenated groups are almost absent or nondetectable. Conversely, for the THF_10d sample, an increase of the signals related to these groups can be observed. This confirms the trend in mass loss visible from the TGA data, in which among the samples treated for 10 days, only THF displays a larger mass loss, indicating a higher concentration of oxygenated groups. This evidence, together with the situation emerging from the Raman data of the same samples, seems to indicate that a prolonged exposure to a mild reaction environment might trigger a reorganization of the structure toward a more ordered situation. Another evidence for such trend is the partial erosion of the band at 1590 cm^–1^: this band intensity is inversely proportional to the extension of sp^2^ conjugation, also evidenced by the increase of the G band in the Raman spectra (see Figure 4).^35^ Such restructuring of the material has already been observed in the past, but with thermal treatment in inert atmosphere (graphitization process).^36^ Therefore, the hypothesis lies in the role of surface energy of the edges of graphitic islands: in fact, oxygenated groups appear to be a “defect” in the regularity of the conjugated sp^2^ structure of graphite, thus possessing a much higher reactivity that makes them perfect anchoring points for interaction with other chemical species.^52^ However, since it has been proven that solvents do not chemically react with the carbon structure, the possible way is a slow rearrangement of the sp^2^ structure leading to an overall growth in conjugation.

Differently from the previous cases, the sample treated for 10 days in THF seems to enhance the trend observed for 3 days of treatment. Indeed, there is an increase of the band at 1715 cm^–1^ due to carbonyl-containing species and the appearance of a new band at 1220 cm^–1^ due the formation of phenolic groups,^53^ also confirmed by the release of CO by solvent-treated samples at a *T* > 1000 K.^45^ This difference might be explained with the temperature of the reaction: the prepared samples were treated at *T* > *T*_boil_ (*T*_boil_ THF = 339 K), meaning that this solvent is in equilibrium with its gas phase. This can be the cause of a much more violent reaction that, under prolonged time, leads to a more disruptive environment for the materials. This is also evident in the trend of the surface area values of the carbons: in fact, the *S*_BET_ of samples modified in THF decreases with time of reaction (see Table 1). Conversely, for the other two solvents, surface area remains almost constant (IPA) or shows an increase (TOL) along with the time of the treatment, thus explaining also the reordering of the structure.

With the aim of a better understanding of the characteristics of samples treated in THF at different contact times, photoelectron spectroscopy was used for assessing surface chemistry before and after solvent treatment and therefore compared to the parent AC.

The survey spectrum reported in Figure S5 indicates that the untreated sample primarily contains carbon (C), oxygen (O), and potassium (K), with other nondetectable impurities. The survey spectra of the samples THF_3d and THF_10d are quite similar, and therefore they are not shown for conciseness; however, the weight percentages (wt %) of detected elements present on the surface are reported in Table 5. A slight increase in oxygen content was observed as a consequence of the solvent treatment, confirming the previously discussed IR data, together with a decrease in potassium content with the solvent contact time.

**Table 5:** Weight Percentage (wt %) of the Species on the Top Surface Obtained by XPS Analysis

**sample**	O 1s (wt %)	C 1s (wt %)	K 2p (wt %)
AC	12.46 ± 0.44	86.29 ± 0.08	1.25 ± 0.52
THF_3d	12.86 ± 1.36	86.30 ± 0.69	0.84 ± 0.57
THF_10d	13.25 ± 0.08	86.32 ± 0.53	0.43 ± 0.22

The fitted C 1s spectra of the investigated samples are shown in Figure 9 (top), and the corresponding deconvolution details are provided in Table 6. All C 1s spectra exhibit a main peak at 284.50 ± 0.08 eV (C1 component), attributed to C=C bonds in graphitic carbon, which is the most abundant species across all samples. The peak at 285.28 ± 0.08 eV (C2 component) corresponds to highly disordered graphite with an sp^3^ bonding configuration.^54^

**Figure 9:**
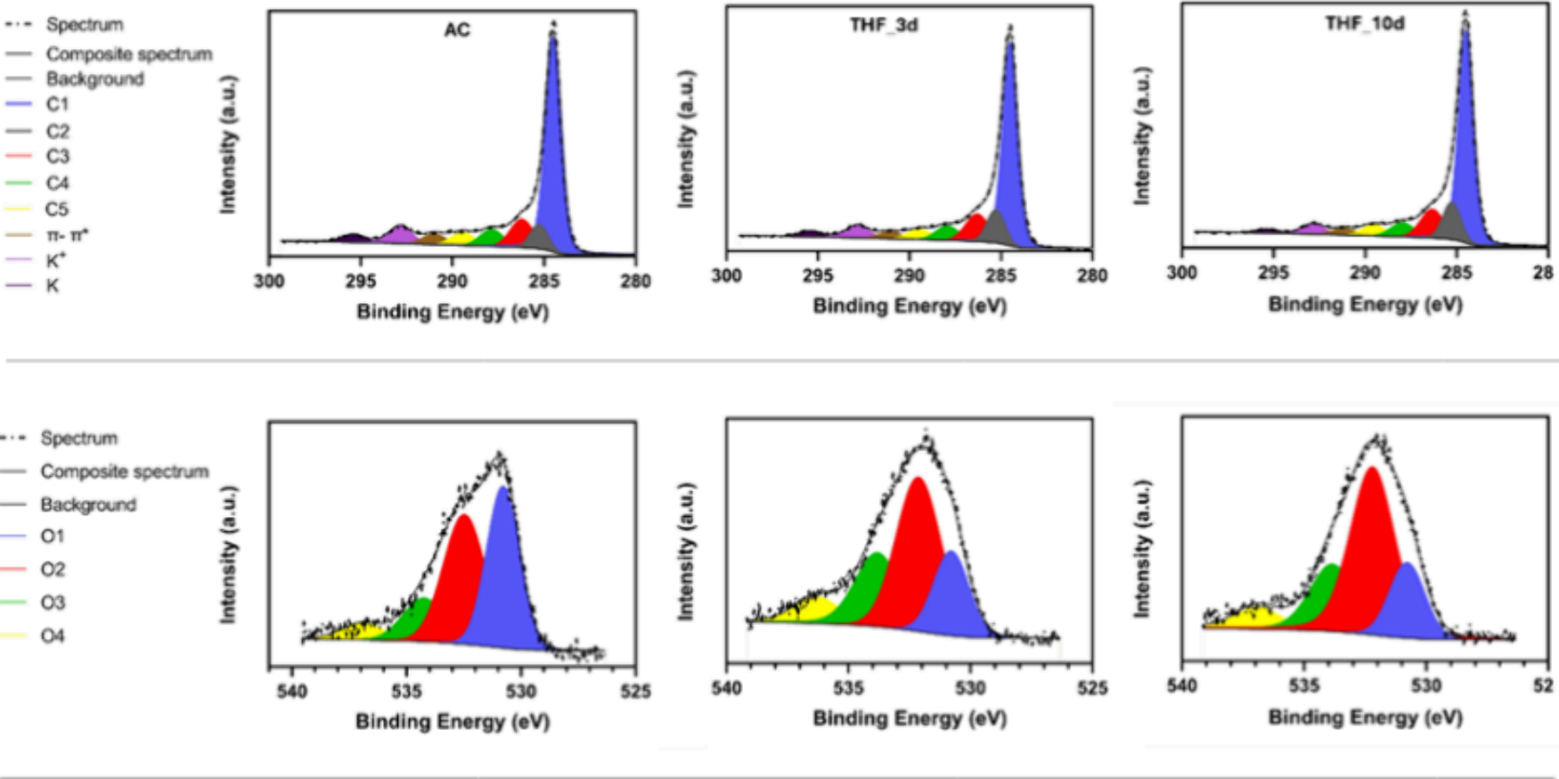
XPS analysis of the investigated samples AC, THF_3d, and THF_10d (top: C 1s; bottom: O 1s).

**Table 6:** Signals for the C 1s Components (BE: Binding Energy; FWHM: Full Width at Half Maximum; Area: Peak Area)

	AC			THF_3d			THF_10d		
species	**B.E.** (eV)	**FWHM**	**area** (%)	**B.E.** (eV)	**FWHM**	**area** (%)	**B.E.** (eV)	**FWHM**	**area** (%)
Sp^2^-C	284.50	1.00	56.05	284.50	1.00	56.96	284.50	1.00	58.59
Sp^3^-C	285.28	1.11	6.67	285.28	1.11	9.88	285.28	1.11	10.75
C–O/C–OH	286.21	1.49	10.81	286.29	1.49	11.36	286.34	1.49	11.53
C=O	287.86	1.49	6.53	287.92	1.49	5.97	287.97	1.49	5.71
COOH/COOR	289.44	1.49	4.96	289.46	1.49	4.28	289.53	1.49	4.47
satellite π–π*	290.99	1.49	4.15	291.04	1.49	3.00	291.13	1.49	2.87
K+	292.84	1.49	7.46	292.84	1.49	5.82	292.84	1.49	4.60
K	295.40	1.49	3.36	295.40	1.49	2.73	295.40	1.49	1.47

A third peak at 286.21 ± 0.18 eV (C3 component) is assigned to C–O species present in cellulose.^55^ The peak at 287.86 ± 0.11 eV (C4 component) is attributed to carbonyl (C=O) groups,^56^ while peaks at 289.44 ± 0.09 eV (C5 component) and 290.99 ± 0.18 eV are ascribed to COOH or carbonates and π–π* shakeup satellite, respectively. Finally, the peaks at 292.84 ± 0.15 and 295.40 ± 0.15 eV are attributed to K^+^ and metallic K, respectively.^27^

All C 1s spectra exhibit similar peak deconvolution. The most notable change is the decrease in the sp^2^/sp^3^ ratio, which drops from 8.40 in the AC sample to 5.76 and 5.45 for THF_3d and THF_10d, respectively. This reduction suggests a slight decrease in long-range order of the materials, accompanied by the formation of more turbostratic and less graphite-like carbon structures.^56^

The O 1s spectrum (Figure 9, bottom) is dominated by oxygen species associated with the carbon material, and it is deconvoluted into four peaks. All signals described for O 1s spectra are summarized in Table 7.^57^

**Table 7:** Signals for the O 1s Components (BE: Binding Energy; FWHM: Full Width at Half Maximum; Area: Peak Area)

	AC			THF_3d			THF_10d		
species	**B.E.** (eV)	**FWHM**	**area** (%)	**B.E.** (eV)	**FWHM**	**area** (%)	**B.E.** (eV)	**FWHM**	**area** (%)
C=O	530.78	1.70	39.88	530.78	1.70	20.23	530.78	1.70	17.46
C–O	532.47	2.16	40.64	532.12	2.16	46.76	532.20	2.16	53.85
–COOH	534.23	2.16	13.62	533.81	2.16	24.15	533.85	2.16	21.59
chemisorbed H_2_O/O_2_	536.91	2.16	5.85	536.29	2.16	8.86	536.91	2.16	7.11

The O1 peak, located at 530.78 ± 0.18 eV, is attributed to carbonyl or quinone species (C=O), while the O2 peak at 532.47 ± 0.14 eV corresponds to C–O bonds. The O3 peak, observed at 534.23 ± 0.23 eV, is assigned to carboxylic groups (COOH/COOR), while the O4 peak, appearing at 536.91 ± 0.16 eV, is related to adsorbed H_2_O or O_2_.

The comparison of O 1s between the AC and solvent-treated samples reveals an increase in the O2 and O3 components at the expense of the O1 peak as a result of solvent treatment, indicating a redistribution of surface oxygen-containing functional groups.

In order to investigate the origin of the oxygen present in the discussed samples treated in THF, an additional test under an inert atmosphere has been carried out since both oxygenated solvents and air atmosphere were used during the abovementioned tests. Therefore, the parent material AC was treated in THF for 3 days under N_2_ flow (THF_3d-N_2_) and then compared to the previously described THF_3d. Also in this case, no significant differences were detected in terms of surface morphology, as shown in the SEM images included in the Supporting Information (Figure S4).

The sample THF_3d-N_2_ shows a similar thermal profile to the correspondent 3-day sample, but some differences in the FT-IR spectra of the gas evolved during the thermal degradation can be observed (Figure 10A). More in detail, the CO_2_ signal is still present in the sample treated under nitrogen, but in smaller quantity. In fact, the ratios between the intensity of the signal at the maximum of the peaks of the CO_2_ stretching (2358 cm^–1^) and C–H stretching (2929 cm^–1^) are equal to 2.4 and 5.8 in THF_3d-N_2_ and THF_3d, respectively. Moreover, the THF_3d-N_2_ carbon shows a split in the C–H stretching signal, with a second maximum at higher wavenumbers (3029 cm^–1^) (Figure 10B). This information concurs with the interpretation that, although a small part of the formation of the oxygenated groups may come from the solvent itself, the most results from the incorporation of atmospheric oxygen during the solvent–carbonaceous matrix contact time. In addition, the solvent action in the absence of atmospheric oxygen creates partial destabilization of the structure, which may exhibit pyrolytic breaks, releasing at high temperatures volatile compounds containing not only alkyl groups (C–H stretching <3000 cm^–1^) but also alkenyl or aromatic groups (C=C stretching >3000 cm^–1^).

**Figure 10:**
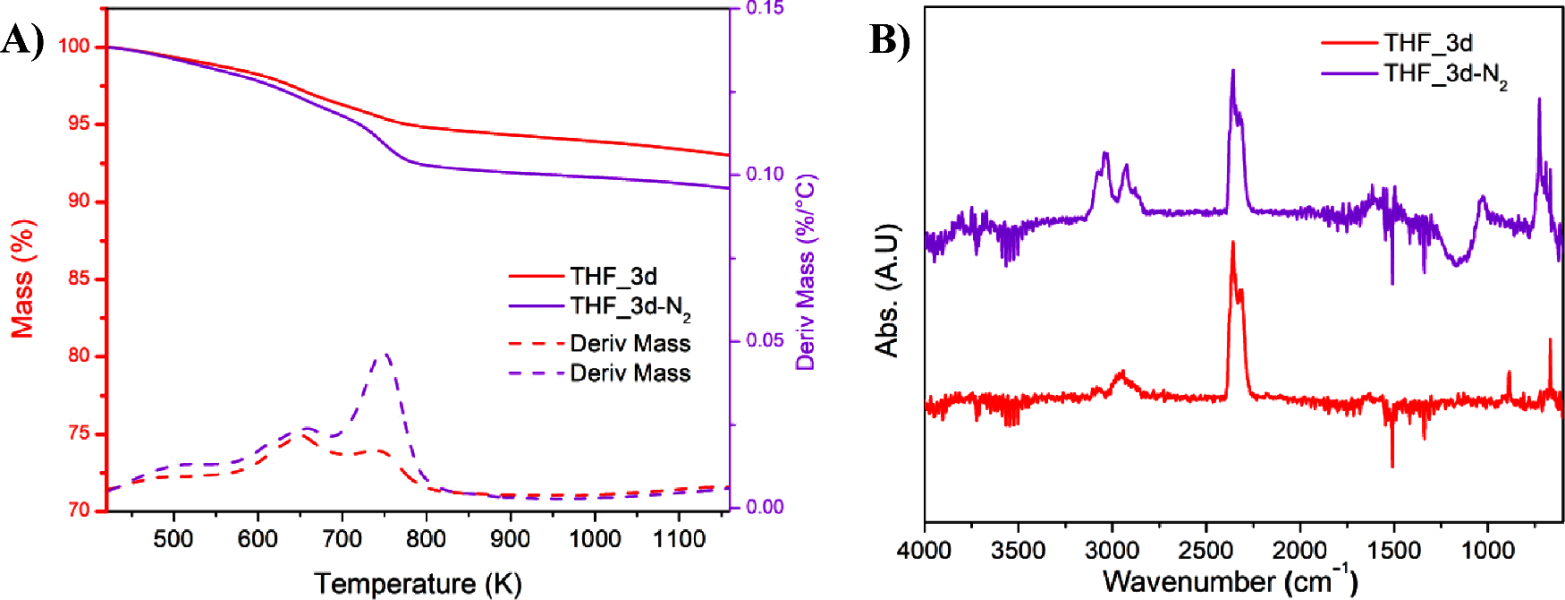
(A) TGA profiles of THF_3d and THF_3d-N_2_ and (B) FT-IR spectra of gas evolved at 523 K for both samples.

The sample treated under inert atmosphere was also analyzed by FT-IR. The obtained data revealed (Figure 11) that when the treatment is performed under nitrogen flow, the rising of the 800 cm^–1^ signal is still observed, indicating the modification of the edges of the graphitic platelets of the carbon; however, the signals generated by the presence of oxygenated groups originated by aromatic rings opening are drastically reduced.

**Figure 11:**
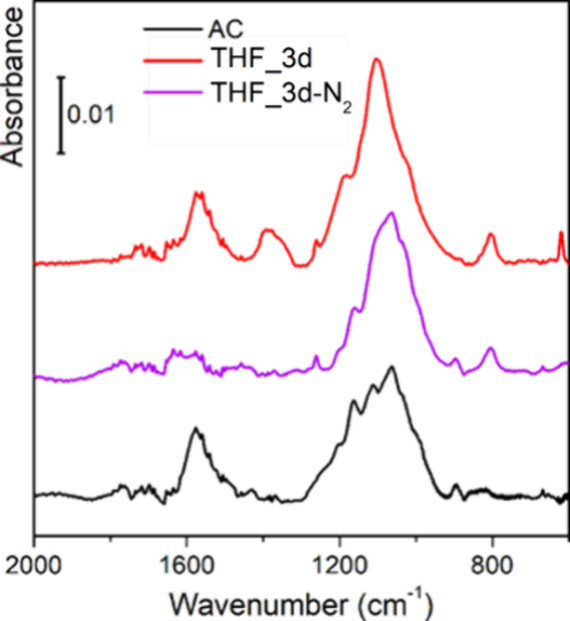
FT-IR spectra of fresh AC (black) and of samples treated for 3 days in THF in air (red), or under N_2_ atmosphere (violet).

These results are also confirmed by Raman spectroscopy and N_2_-physisorprtion analyses. In fact, as reported in Table 8, the *I*_D_/*I*_G_ ratio of THF_3d-N_2_ shows a slightly higher value than the sample prepared in oxidizing atmosphere THF_3d (see Figure S5), indicating a partial increase in the degree of disorder mainly ascribable to a higher content of sp^3^ carbon with respect to the sp^2^ one. In addition, while the treatment under air led just to a reduction of about 9% in *S*_BET_, compared to the parent sample AC, THF_3d-N_2_ reveals a 30% drop. Finally, significant differences were also observed in terms of pore volume with respect to the parent AC sample, in particular a reduction of the total pore volume (−4% in THF_3d versus −20% in THF_3d-N_2_,), and a drastic decrease in micropore volume (≈69%), confirming the possibility of some pyrolytic breaks occurring and the general reduction in the framework stability. However, it is not feasible to establish a direct correlation between the relatively small changes in the Raman spectra with such a large difference in the textural properties evidenced by BET analyses.

**Table 8:** Physicochemical Properties of Samples Treated in THF for 3 Days under N_2_ or an Air Atmosphere

**sample**	***I***_**D**_ **/*I***_**G**_ ^a^	***S***_**BET**_^b^ (m^2^/g)	***V***_**micro**_^b^ (cm^3^/g)	***V***_**TOT**_^b^ (cm^3^/g)	**mass loss^c^%** (423–573 K)	**mass loss**^c^**%** (573–873 K)	**mass loss**^c^**%** (873–1173 K)	**residue**^c^**%** (>1173 K)
AC	1.05	1065	0.396	0.405	1.0	1.4	2.4	95.3
THF_3d	1.10	973	0.380	0.387	1.5	4.1	1.6	92.8
THF_3d-N_2_	1.16	744	0.121	0.324	1.8	6.2	1.2	90.8

a Measured by Raman spectroscopy.

b Measured by N_2_-physisorption analysis at 77 K.

c Measured by TGA under N_2_ in the RT–1173 K temperature range.

### Hydrogen Uptake Preliminary Evaluation

Hydrogen adsorption and desorption isotherms at RT (here not shown) and 77 K up to a maximum pressure of 1 bar have been performed on all of the prepared ACs. All samples show negligible adsorption at RT, while interesting uptake values can be observed at liquid nitrogen temperature. Figures 12 and 13 below show H_2_ adsorption–desorption isotherms acquired on AC materials treated with different solvents for 3 and 10 days, respectively.

**Figure 12:**
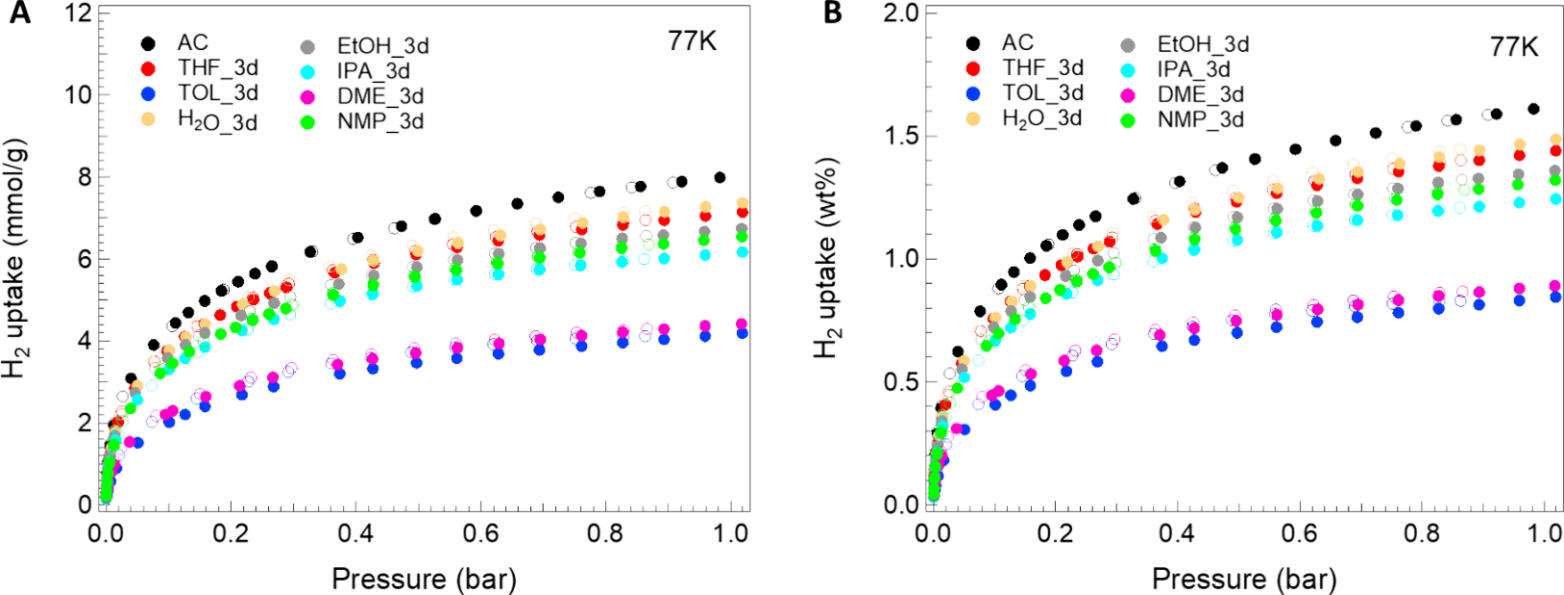
H_2_ adsorption (full dots) and desorption (empty dots) isotherms up to 1 bar and temperature of 77 K for samples treated with different solvents for 3 days, reported as (A) mmol/g and (B) wt.%.

**Figure 13:**
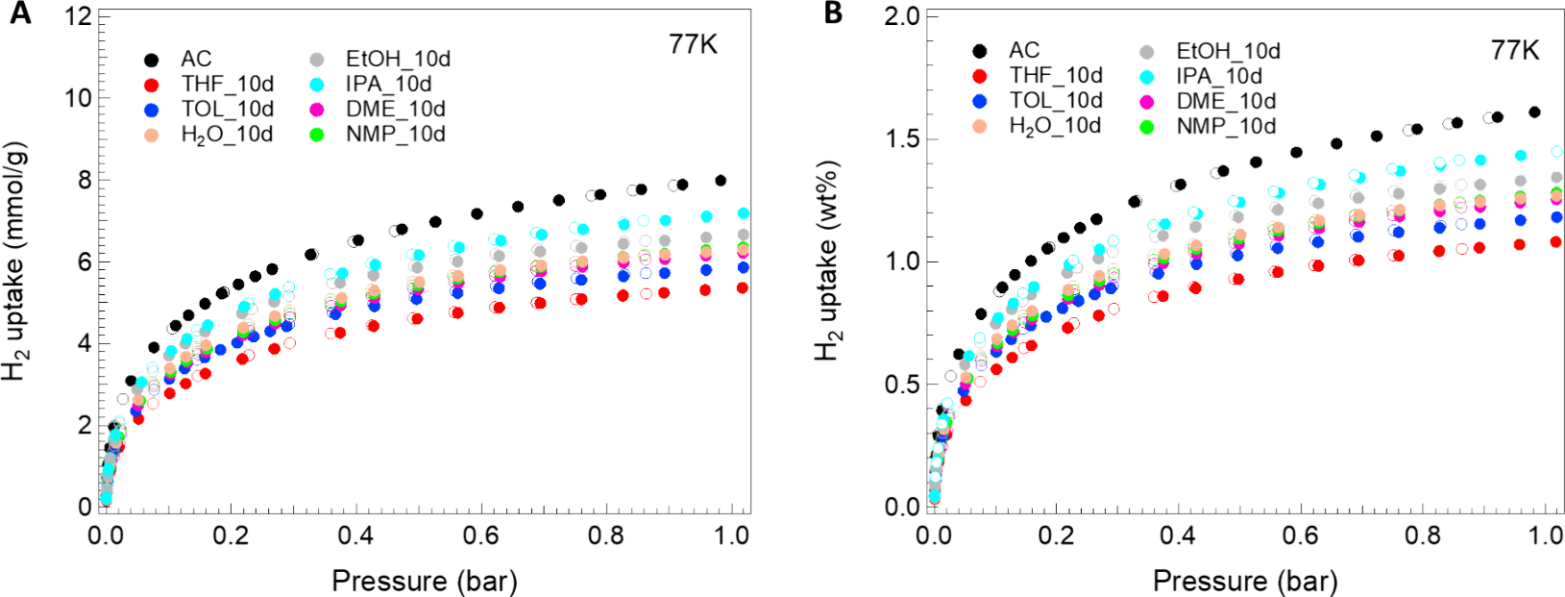
H_2_ adsorption (full dots) and desorption (empty dots) isotherms up to 1 bar and temperature of 77 K for samples treated with different solvents for 10 days, reported as (A) mmol/g and (B) wt.%.

For all of the prepared samples, it can be noted that the maximum adsorption capacity appears lower compared to the parent AC used as raw materials for each treatment process. In fact, the parent AC sample shows a maximum adsorption of 7.99 mmol/g (∼1.61 wt.%) while all other samples show uptake value at most of 7.37 mmol/g (∼1.49 wt.%) (see Table 6).

Looking at the different adsorption isotherms in detail, it is evident that all curves show a similar trend. A common characteristic is, for example, the storage capacity between 2 and 4 mmol/g reached at pressures below 0.1 bar by all samples, together with a minimum uptake value of almost 4.19 mmol/g reached at the maximum analyzed pressure and a fully reversible adsorption process. The fast and high adsorption of H_2_, which occurs in the pressure range 0–0.3 bar, is typical of materials with a high degree of microporosity with preferential filling of ultramicropores and then supermicropores.^58^ At *P*/*P*_0_ > 0.3, pores straddling the 2 nm gradually fill up, reaching the maximum adsorption capacity at the measuring pressure.

The behavior just described, combined with the complete reversibility of the adsorption/desorption process, is maintained by all samples demonstrating how the H_2_ adsorption capacity depends exclusively on the material’s porous structure and on the molecule–surface interaction. The absence of hysteresis phenomena in the isotherms demonstrates how the treatment with the various solvents mainly brings structural changes. The adsorption properties of the samples reflect and are consistent with the textural properties obtained after the treatment with the different solvents, which, as previously described, have brought about changes either in the reduction of the specific surface area available to the gas or in the size of the pores. The noteworthy aspect is that these textural changes vary based on the solvent used and, in some cases, have little impact on the carbonaceous structure or allow a rearrangement of the same after a certain time. In this regard, both H_2_O and THF 3-day samples and IPA at 10 days show good results in terms of showing only a slight decrease in H_2_ adsorption compared to the parent AC.

Analyzing the results based on the duration of treatment, the gap between the maximum and minimum uptake value for each analyzed solvent shows a clear trend, in particular a reduction from a difference of 3.18 mmol/g for the 3-day treatment until a value of 1.83 mmol/g for longer exposure (10 days). Although the variations in the solvent/days combo do not follow a well-defined trend, for a few of them, this can be observed. In fact, the DME and TOL solvent show an increase in the maximum H_2_ adsorption uptake (∼40%) by increasing the solvent–matrix contact time while, in contrast, the H_2_O one exhibits a decrease of 15% moving from 3 to 10 days of treatment.

Focusing on the solvents of greatest interest (TOL, IPA, and THF), the trends observed in maximum adsorption capacities (see Table 9) reflect those previously reported for structural properties. In particular, samples treated with TOL show an increase in storage capacity at the maximum analyzed pressure (1 bar) of 39% with increasing days of treatment, and similarly, samples treated with IPA show an increase of 17%. In contrast, according to the previous-described framework changes, the sample treated with THF shows a reduction in maximum adsorption capacity of 25% with increasing the time.

**Table 9:** Maximum H_2_ Uptake Values for All Analyzed Samples

**sample**	**H**_**2**_**uptake**^a^ (mmol/g)	**H**_**2**_**uptake**^a^ (wt %)
AC	7.99	1.61
THF_3d	7.14	1.44
THF_10d	5.36	1.08
TOL_3d	4.19	0.85
TOL_10d	5.86	1.18
DME_3d	4.42	0.89
DME_10d	6.22	1.25
NMP_3d	6.54	1.32
NMP_10d	6.36	1.28
H_2_O_3d	7.37	1.49
H_2_O_10d	6.29	1.27
EtOH_3d	6.74	1.36
EtOH_3d	6.67	1.34
IPA_3d	6.17	1.24
IPA_10d	7.19	1.45

a Performed at 77 K within a pressure range of 0–1 bar.

Finally, Figure 14 shows the correlation between adsorption capacity and structural parameters, calculating the relative correlation coefficient. In particular, it can be easily observed that specific surface area and total pore volume are the parameters that mainly influenced the H_2_ uptake, as demonstrated by a higher correlation coefficient (see Figure 14A,F). It is also noteworthy that, within the total pore volume, an important contribution to the mentioned correlation is due to the micropore fraction showing a correlation close to 0.92 and higher than that shown by the mesopore one (0.06).

**Figure 14:**
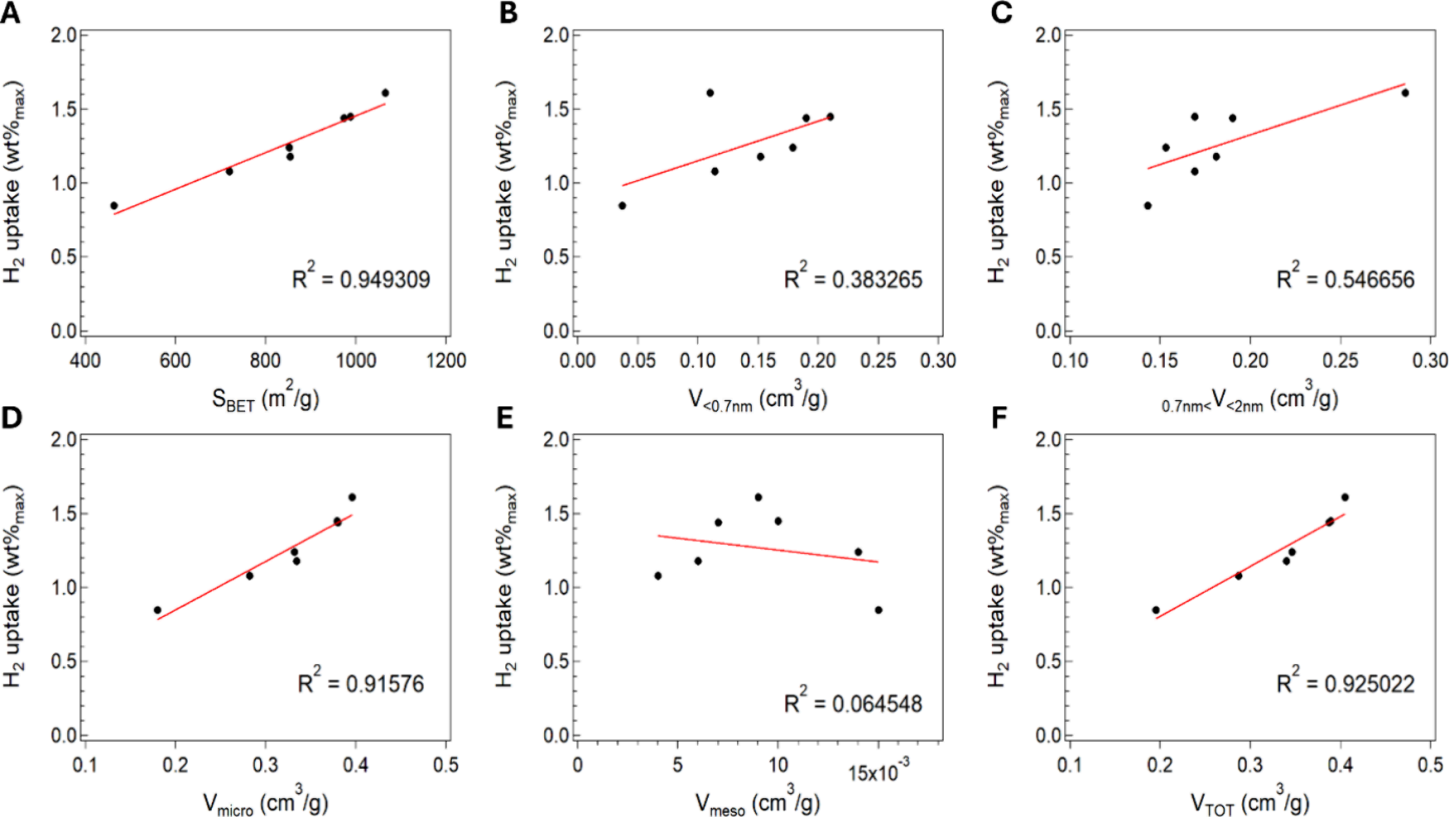
Correlation between the maximum hydrogen uptake at 1 bar and 77 K and (A) BET surface area, (B) ultramicropore volume, (C) supermicropore volume, (D) micropore volume, (E) mesopore volume, and (F) total pore volume, respectively.

## Conclusions

In this work, the effect of several solvents on the biomass-derived activated carbon framework was investigated at three different contact times, i.e., 3, 7, and 10 days, and under mild reaction conditions, with the ultimate goal of identifying the optimal setting for liquid-phase carbon doping/functionalization in hydrogen storage applications.

Changes in textural properties of the prepared samples were evaluated, resulting in a general decrease in either specific surface area or micropores volume compared to the parent AC. However, certain specific solvents, such as toluene, 1,2-dimethoxyethane, water, and isopropyl alcohol led to an enhancement of the structural order at longer contact times, indicating a framework rearrangement. This has been attributed to the fact that, being activated carbon metastable materials, the used reaction conditions could push the carbonaceous matrix toward its thermodynamic minimum, i.e., a graphite-like structure.

On the contrary, samples treated with tetrahydrofuran revealed a drop in the textural properties, as well as in the thermal stability as a function of the reaction time. In this case, the fact of having used a reaction temperature higher than the boiling point of THF led to a much more disruptive environment for the activated carbons.

Although it is not possible to identify a general trend in terms of contact time and/or solvent, the deeper characterization of the selected samples (with TOL, THF, and IPA at 3 and 10 days), together with the test carried out under an inert atmosphere (THF_3d-N_2_), allowed us to determine also the role of oxygen in affecting the carbonaceous framework. In fact, it has been shown that the presence of atmospheric oxygen results in the formation of oxygenated functional groups on the carbon surface, which are absent in the case of ACs treated under nitrogen. Indeed, the insertion of O-containing functional groups seems to be necessary for carbon structural rearrangement toward a graphite-like situation when exposing the samples to a reaction environment for a prolonged time.

Finally, the preliminary evaluation of hydrogen uptake at 77 K carried out on all prepared samples has shown that the maximum adsorption capacity is lower than that of the parent AC in all cases; however, it is noteworthy that the storage capacity increases between 17 and 39% by increasing the contact time in the presence of isopropyl alcohol and toluene, respectively. On the contrary, THF as solvent led to a 25% reduction of the maximum adsorption capacity with increasing time, reflecting the demonstrated direct correlation between structural parameters and adsorption capacity, in particular referring to specific surface area, micropores volume, and total pore volume.

## Supplementary Material



## Notes

SEM images of prepared samples (Figures S1 and S4); thermal profiles (Figure S2); FT-IR (Figure S3); and survey spectrum (Figure S5) (PDF)
