# Supplementary material for: Solvent Effects in Biomass-Derived Activated Carbons: New Insights for Their Doping/Functionalization toward Potential Hydrogen Storage Applications
Source: Langmuir. 2025 May 14;41(20):12619–33. doi: 10.1021/acs.langmuir.5c00711 (PMC12120978; doi:10.1021/acs.langmuir.5c00711)

SUPPORTING INFORMATIONS

SOLVENT EFFECTS IN BIOMASS-DERIVED ACTIVATED CARBONS: NEW INSIGHTS FOR THEIR DOPING/FUNCTIONALIZATION TOWARD POTENTIAL HYDROGEN STORAGE APPLICATIONS

Alessia MARINO[1,2], Carlo POSELLE BONAVENTURA[3], Sara SCIARRETTA[1], Giuseppe CONTE[3], Chiara PELOSI[4], Andrea LAZZARINI[1,2], Alfredo ALOISE[1,2], Celia DUCE[4], Luca BERNAZZANI[4], Alfonso POLICICCHIO[3,5,6,*], Marcello CRUCIANELLI[1,2,*]

Affiliations

[1]Department of Physical and Chemical Sciences, University of L'Aquila, Via Vetoio, I-67100 L'Aquila

[2]Consorzio Interuniversitario Nazionale per la Scienza e Tecnologia dei Materiali (INSTM), Via Giuseppe Giusti 9, I-50121 Firenze

[3]Department of Physics, Università della Calabria, Via Pietro Bucci cubo 31C, I-87036 Arcavacata di Rende (CS)

[4]Department of Chemistry and Industrial Chemistry, University of Pisa, Via G. Moruzzi 13, I-56124 Pisa

[5]CNISM - Consorzio Nazionale Interuniversitario per le Scienze fisiche della Materia, Via della Vasca Navale 84, I-00146 Roma

[6]Consiglio Nazionale delle Ricerche, Istituto di Nanotecnologia (Nanotec)-UoS Cosenza, Via Ponte P. Bucci, Cubo 31C, I-87036 Arcavacata di Rende (CS)

Nº of pages: 6

Nº of figures: 5

Nº of tables: 1

Table S1. Elemental analysis CHNS/O of samples treated with selected solvents for 3 and 10 days.

Sample	%C	%H	%N	%S	%O*
**AC**	85.25	0.69	0.00	0.05	14.01
**TOL_3d**	85.91	0.71	0.00	0.03	13.35
**TOL_10d**	85.07	0.83	0.00	0.04	14.06
**IPA_3d**	86.11	0.77	0.00	0.03	13.09
**IPA_10d**	86.02	0.75	0.00	0.02	13.21
**THF_3d**	85.24	0.74	0.00	0.04	13.98
**THF_10d**	85.03	0.79	0.00	0.05	14.13

*Calculated by difference

Figure S1. SEM images of the prepared samples at different magnifications: **a)** THF_3d at mag. 10000 x; **b)** THF_3d at mag. 10000 x; **c)** and **d)** AC at mag. 10000 x and 20000 x, respectively; **e)** and **f)** IPA_3d at mag. 10000 x and 20000 x, respectively; **g)** and **h)** IPA_10d at mag. 10000 x and 20000 x, respectively; **i)** and **l)** TOL_3d at mag. 10000 x and 20000 x, respectively; and **m)** and **n)** TOL_10d at mag. 10000 x and 20000 x, respectively.

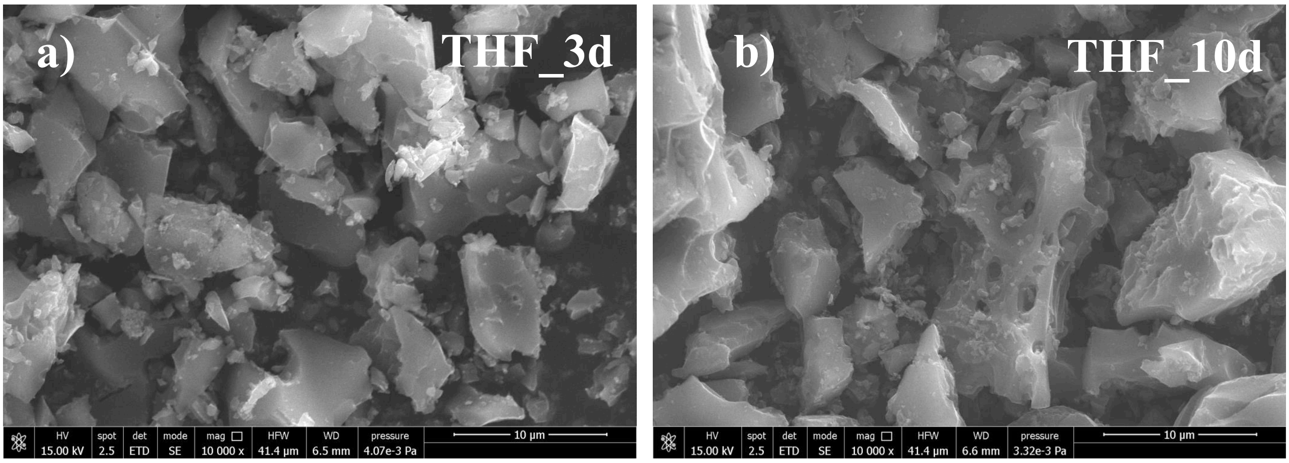

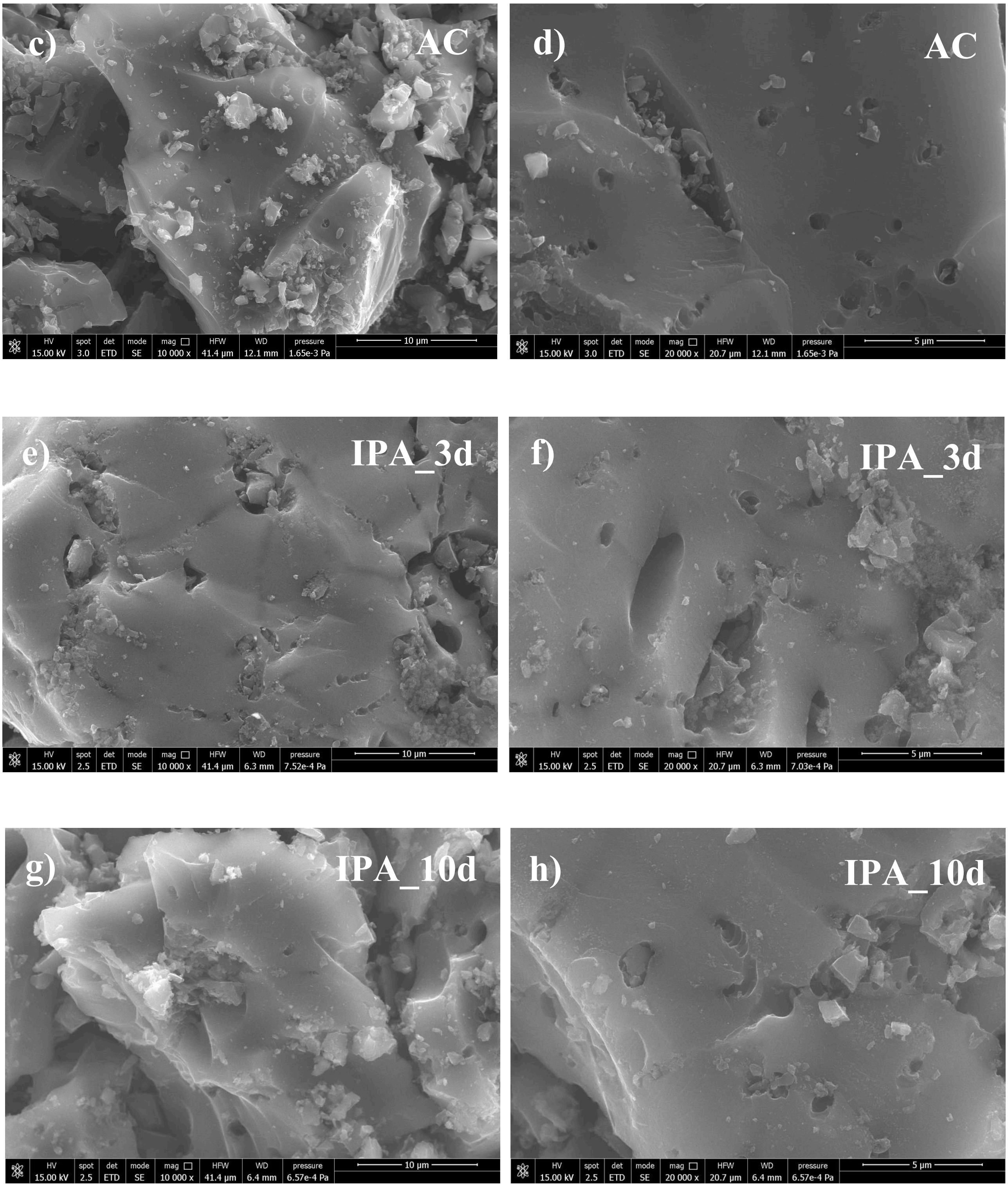
c)
AC
10 µm
d)
AC
5 µm
e)
IPA_3d
10 µm
f)
IPA_3d
5 µm
g)
IPA_10d
10 µm
h)
IPA_10d
5 µm

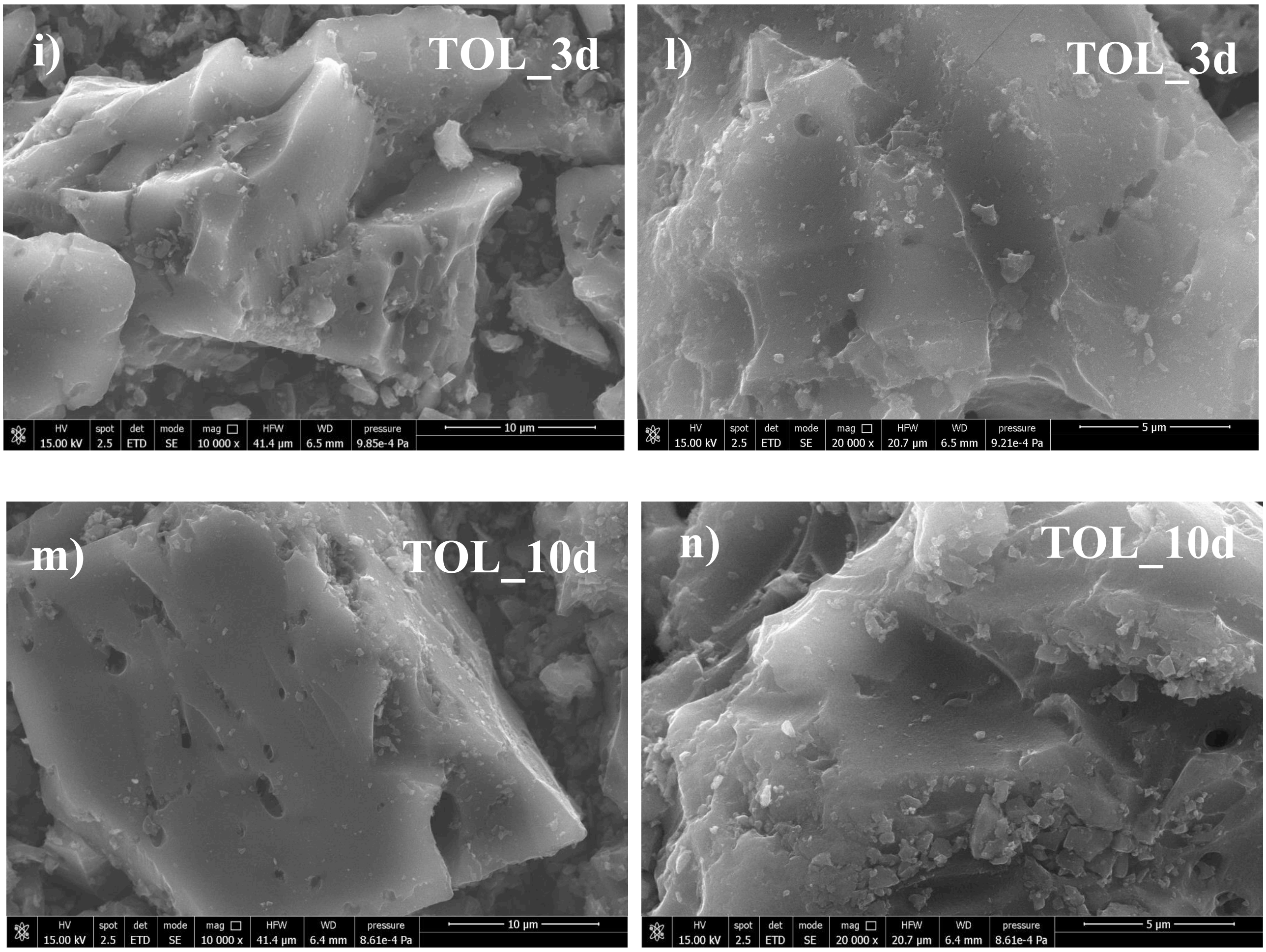

Figure S2. Complete thermal profiles of the samples treated in THF (a), TOL (b), and IPA (c).

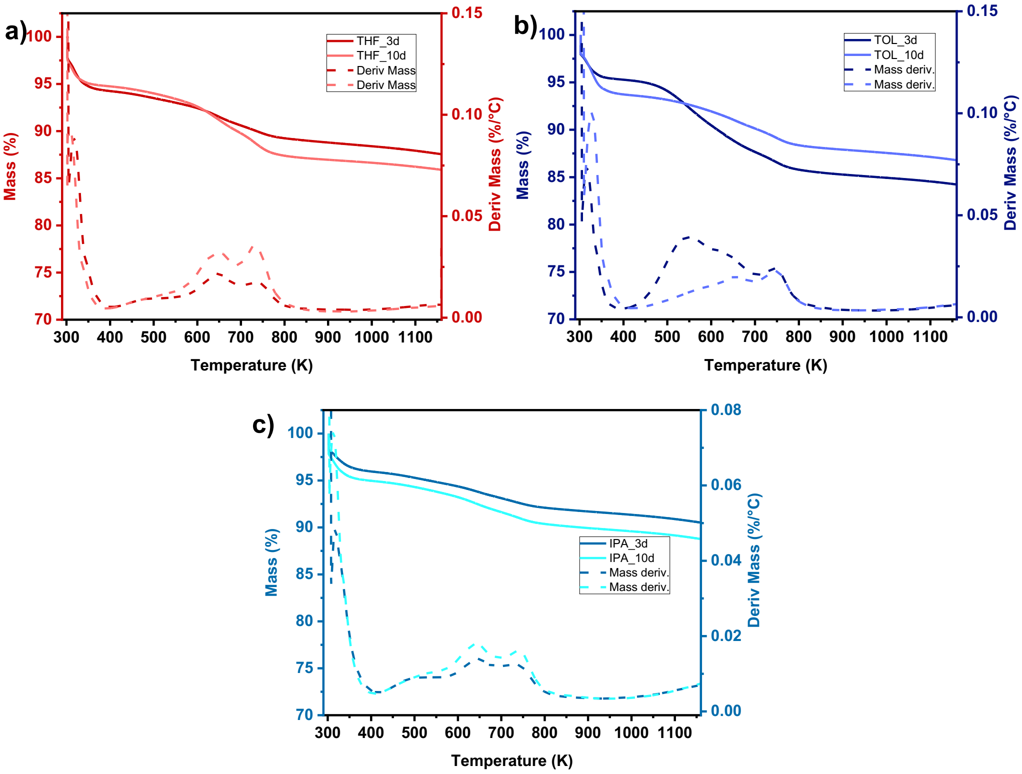

Figure S3. FT-IR spectra of gas evolved at 700 K for **a)** THF_3d, **b)** THF_10d, **c)** TOL_3d, **d)** TOL_10d, **e)** IPA_3d, **f)** IPA_10d. g) FT-IR spectrum of gas evolved at 1000 K for THF_10d.

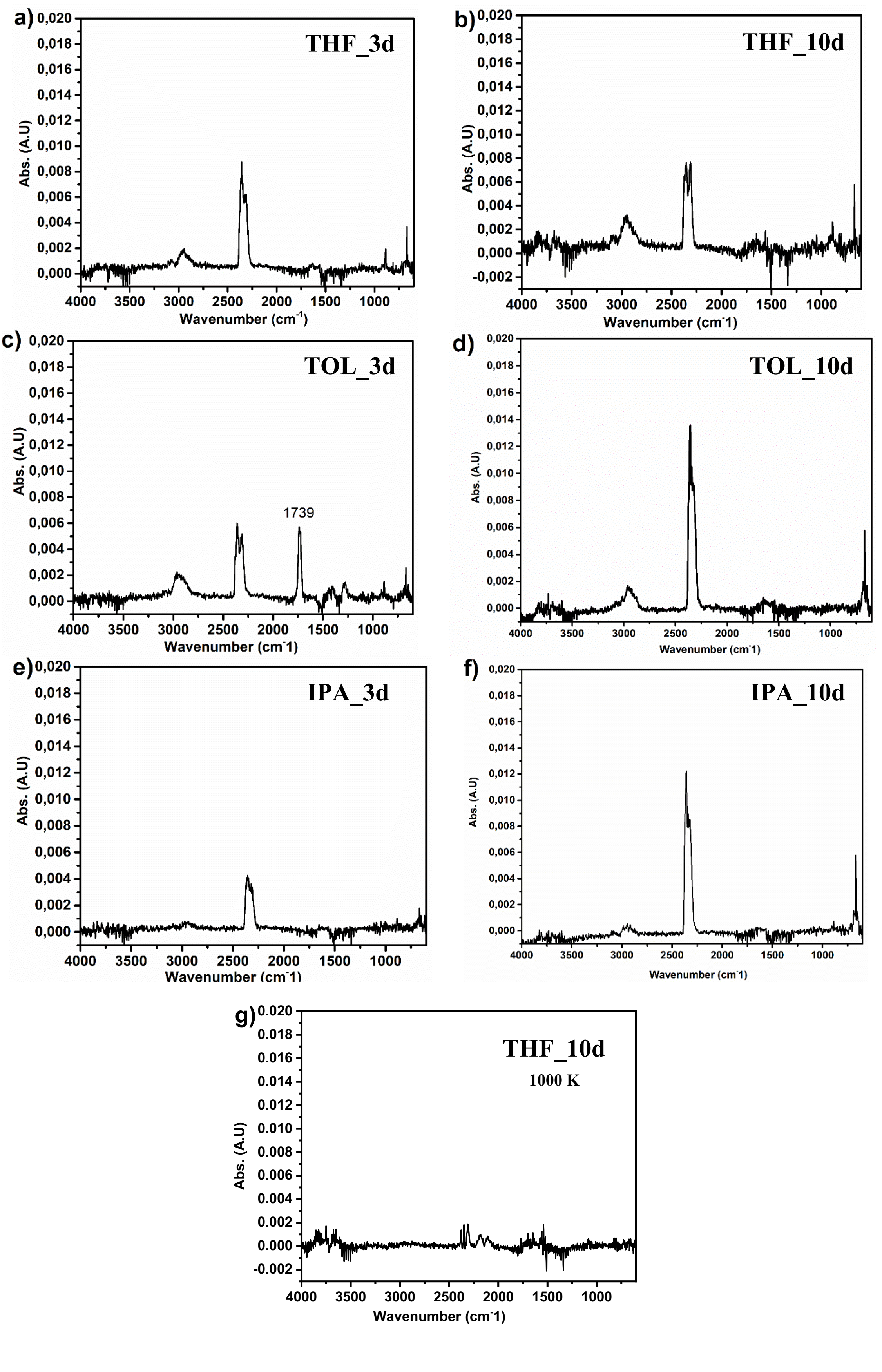

Figure S4. SEM images of the sample THF_3d-N_2 at **a)** mag. 10000 x and **b)** mag. 20000 x.

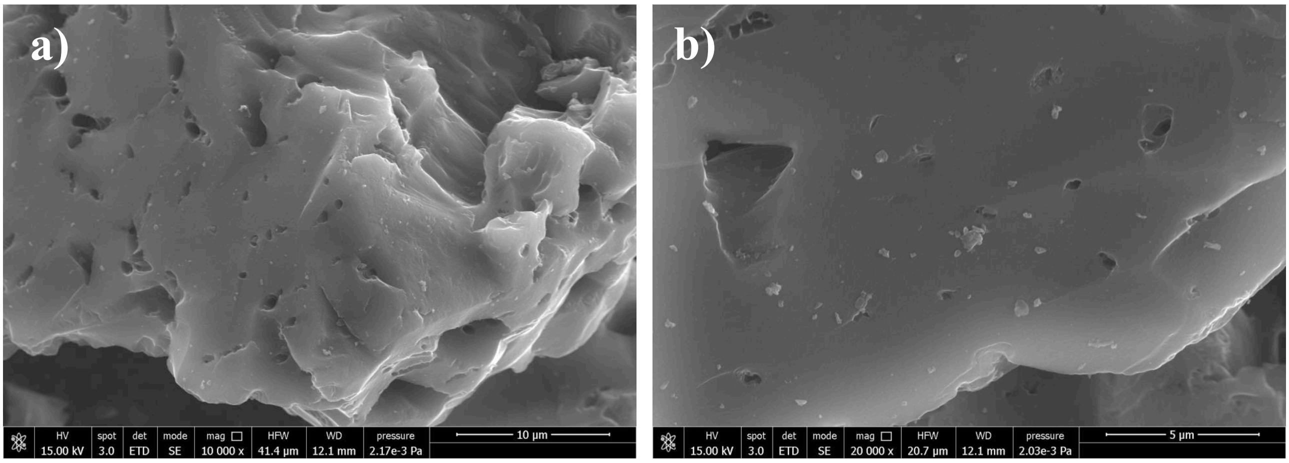

Figure S5. Survey spectrum of the untreated AC sample.

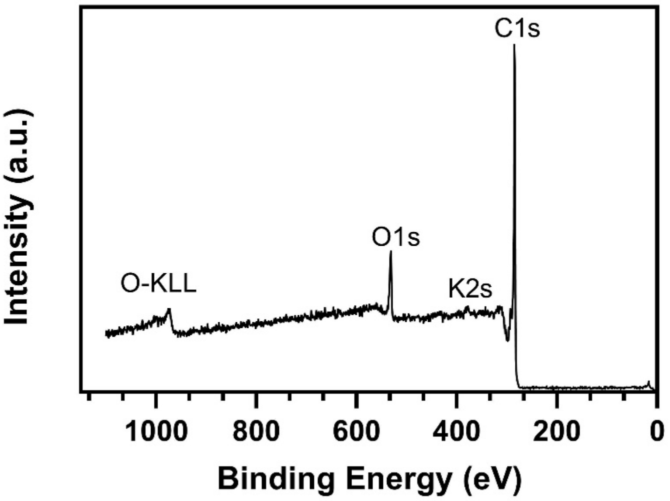

Supplement: Supplementary file 1 [file la5c00711_si_001.pdf]
